# All brains are made of this: a fundamental building block of brain matter with matching neuronal and glial masses

**DOI:** 10.3389/fnana.2014.00127

**Published:** 2014-11-12

**Authors:** Bruno Mota, Suzana Herculano-Houzel

**Affiliations:** ^1^Instituto de Física, Universidade Federal do Rio de JaneiroRio de Janeiro, Brazil; ^2^Instituto Nacional de Neurociência TranslacionalSão Paulo, Brazil; ^3^Instituto de Ciências Biomédicas, Universidade Federal do Rio de JaneiroRio de Janeiro, Brazil

**Keywords:** allometry, glia/neuron ratio, number of neurons, number of glial cells, cell size, brain size

## Abstract

How does the size of the glial and neuronal cells that compose brain tissue vary across brain structures and species? Our previous studies indicate that average neuronal size is highly variable, while average glial cell size is more constant. Measuring whole cell sizes *in vivo*, however, is a daunting task. Here we use chi-square minimization of the relationship between measured neuronal and glial cell densities in the cerebral cortex, cerebellum, and rest of brain in 27 mammalian species to model neuronal and glial cell mass, as well as the neuronal mass fraction of the tissue (the fraction of tissue mass composed by neurons). Our model shows that while average neuronal cell mass varies by over 500-fold across brain structures and species, average glial cell mass varies only 1.4-fold. Neuronal mass fraction varies typically between 0.6 and 0.8 in all structures. Remarkably, we show that two fundamental, universal relationships apply across all brain structures and species: (1) the glia/neuron ratio varies with the total neuronal mass in the tissue (which in turn depends on variations in average neuronal cell mass), and (2) the neuronal mass per glial cell, and with it the neuronal mass fraction and neuron/glia mass ratio, varies with average glial cell mass in the tissue. We propose that there is a fundamental building block of brain tissue: the glial mass that accompanies a unit of neuronal mass. We argue that the scaling of this glial mass is a consequence of a universal mechanism whereby numbers of glial cells are added to the neuronal parenchyma during development, irrespective of whether the neurons composing it are large or small, but depending on the average mass of the glial cells being added. We also show how evolutionary variations in neuronal cell mass, glial cell mass and number of neurons suffice to determine the most basic characteristics of brain structures, such as mass, glia/neuron ratio, neuron/glia mass ratio, and cell densities.

## Introduction

Brain tissue is composed of neuronal, glial and endothelial cells, and although there must be biological rules that determine the numbers of cells of each subtype and the volumes (or masses) occupied by them, little is known about such rules, if they indeed exist. It is now acknowledged that neuronal function depends tightly on various aspects of glial cell physiology (Barres, 2008), which raises the possibility that precise relationships between numbers, or volumes, of neuronal and glial cells must exist in the brain. Yet neuroscience still does not know the basics of brain tissue construction: For instance, what proportion of brain tissue volume consists of neurons, and what proportion consists of glial cells? Is this proportion variable across brain structures and species, or fairly constant? How is this most basic property of brain tissue determined?

Neurons are the smallest separable units of information processing in brains. Their extended shapes, with processes that can reach lengths of many multiples of the somatic diameters, results in much of their masses being distributed non-locally. This non-local distribution of neurons and their arbors makes it nearly impossible to estimate directly the cellular mass of neurons and the fraction of tissue volume compose by them. Similarly, little is known about the proportion of brain tissue that is composed of glial cells; how large they are compared to neurons (beyond simple soma size), or how the average size of glial cells varies across structures and species. Glial cells were once supposed to vastly outnumber neurons, especially in large brains such as the human brain, but now that is known not to be the case (Azevedo et al., 2009). Endothelial cells, in contrast, are presumed to be relatively few, given that the relative volume of the vasculature ranges between only 2 and 4% of brain volume in the human brain (Lawers et al., 2008), 5–6% in the rat brain (Buchweitz and Weiss, 1986), 1% in the mouse cortex (Tsai et al., 2009), and does not correlate significantly with brain size across mammalian species, varying around 2% of brain volume (Karbowski, 2011).

The development of the isotropic fractionator (Herculano-Houzel and Lent, 2005) made feasible the precise measurement of total numbers of neuronal and non-neuronal cells in brain structures, leading the way to comparative studies of brain cellular composition across mammalian orders, including humans (Herculano-Houzel et al., 2006, 2007, 2011; Azevedo et al., 2009; Sarko et al., 2009; Gabi et al., 2010). These studies have shown that mammalian brain structures scale in size as different power functions of their numbers of neurons across orders (reviewed in Herculano-Houzel, 2011a). For instance, the cerebral cortex and cerebellum of primates, and the cerebellum of insectivores, vary in size across species as nearly linear functions of their numbers of neurons, such that neuronal densities change very little accompanying structure size. In contrast, the cerebral cortex and cerebellum in rodent brains as well as the cerebral cortex of eulipotyphlans increase in size as steep power functions of their numbers of neurons, with neuronal densities that decrease markedly with increasing structure size. On the other hand, all brain structures of all the mammalian species analyzed so far scale in mass according to similar, nearly-linear functions of their numbers of non-neuronal cells, which, given the small relative volume of brain vasculature (Lawers et al., 2008), presumably consist predominantly of glial cells. This relationship between structure mass and number of non-neuronal (glial) cells is shared across brain structures and mammalian species of primates, glires, eulipotyphlans, afrotherians, and artiodactyls (Herculano-Houzel et al., 2006, 2007, 2011; Azevedo et al., 2009; Sarko et al., 2009; Gabi et al., 2010; Neves et al., 2014; Kazu et al., 2014). As a consequence, non-neuronal (glial) cell density varies little across orders and structures; but the little variation there is turns out to be correlated with the (much larger) variation in neuronal density. This uniformity in the relationship between brain structure mass and number of non-neuronal, presumably glial, cells is the fundamental observation upon which the present study is based.

Given the great variability in neuronal densities and the small variation in non-neuronal densities across brain structures and species (reviewed in Herculano-Houzel, 2011a), average neuronal cell size (including all dendritic and axonal arbors) is presumed to vary greatly across structures and species, while average glial cell size is presumed to very little. However, because the tissue is composed of neuronal and non-neuronal cells in volumetric proportions currently unknown, the inverse of cell density serves only as an upper bound for the average cell size for each cell type and of the cubic root of the distance across cell bodies of either type, but cannot be construed as a proper estimate of average cell size. If we could determine directly how the average mass of the neuronal and glial cells themselves scale with brain size (or numbers of these cells) for different orders and brain structures, and estimate the mass fraction corresponding to neurons therein, we would be able to fully characterize the scaling of brain structures at a histological level, and understand the origin of the intriguing uniformity in non-neuronal scaling rules (Herculano-Houzel, 2011a). In particular, it would be possible to determine if any of these structural features of brain tissue are universal; to identify others that are contingent to different orders and structures; and to derive explicitly the size and composition of each brain structure for each species analyzed from a small number of scaling rules (some of them universal, and others order- or structure-dependent).

Unfortunately, while one can count cells of each type and measure overall structure mass, one cannot directly weigh individual cells to determine the average neuronal and non-neuronal cell mass—and reconstructing entire cells and their arbors in the different brain structures of a variety of species would be a daunting task. However, as we demonstrate here, it is possible to postulate biologically plausible, simple relations for the scaling of both the average glial cell mass and the glial mass fraction of each structure. As described above, the problem in estimating average cell sizes from the inverse of cell densities is that (1) neuronal and glial cell densities could vary independently of each other, and (2) the mass (or volume) fraction occupied by neurons and glial cells is unknown. Considering that neuronal and glial cell densities are actually correlated, and given a simple set of mathematical relationships among these parameters and the glial and neuronal mass fractions, we arrive at a model relating neuronal and glial cell densities, quantities that have been measured. We then use chi-square minimization to find the model parameters that yield the best fit between the theoretical and the empirical relations obtained for 27 mammalian species, which in turn allows us to estimate the average neuronal and glial cell masses, as well as the fraction of tissue mass that is composed of neurons and of glia (the neuronal mass fraction f_N_ and the glial mass fraction f_G_), for each brain structure and each species analyzed. This is, to our best knowledge, the first time these quantities are estimated systematically.

## Materials and methods

All data analyzed here have been published previously, and consist of mass of and numbers of neuronal and non-neuronal cells found in the whole cerebral cortex (including white matter), whole cerebellum (including white matter and deep cerebellar nuclei) and rest of brain (the ensemble of brainstem, diencephalon, and striatum) of a total of 27 species: nine rodent species (Herculano-Houzel et al., 2006, 2011, excluding the naked mole-rat), twelve primate species, including humans, and one scandentia (Herculano-Houzel et al., 2007; Azevedo et al., 2009; Gabi et al., 2010), and five eulipotyphlan species (Sarko et al., 2009). All experiments were conducted in accordance with US and Brazilian guidelines regarding the use of animals and human subjects in research. All values refer to species averages, which guarantee that analyses regard relationships across species only, and are not confounded by intraspecific variability.

All numbers of cells were obtained with the same method, the isotropic fractionator (Herculano-Houzel and Lent, 2005), which yields results that are similar to those obtained with stereology (Bahney and von Bartheld, 2014; Miller et al., 2014). The isotropic fractionator consists of mechanically dissociating the fixed and dissected structures in a saline detergent solution, such that cell membranes are disrupted, but nuclear membranes remain intact. This allows the free cell nuclei to be collected in a suspension of known volume that is made isotropic by agitation. Samples of the suspension are counted in a hemocytometer to determine the number of nuclei per unit volume of the suspension, and in the total suspension volume. This is referred to as the total number of cells in the structure.

The total number of neurons is then determined by applying to the total number of cells the percentage of nuclei that express NeuN, a neuronal-specific antigen that is found in all cortical and cerebellar neurons, to the exception of Purkinje cells (Mullen et al., 1992). These, however, represent such a small minority of all cerebellar neurons (between 1/700 and 1/3000 granule cell neurons for the species analyzed here; Lange, 1975) that the number of neurons estimated in the cerebellum is not significantly affected by their exclusion from the pool of NeuN-labeled, neuronal nuclei. In the cerebral cortex, NeuN labeling has been shown to give similar quantitative results as morphological analysis of cresyl violet-stained cells, being particularly useful in distinguishing small neurons from glia (Gittins and Harrison, 2004). Additionally, because labeled nuclei are identified by visual inspection under the microscope and not by automated methods, we could confirm that all NeuN-labeled nuclei in each sample were indeed of neuronal morphology and that all nuclei of a particular labeled morphology were labeled in the sample.

Numbers of non-neuronal cells are derived by subtraction of NeuN-labeled nuclei from total numbers of nuclei. Non-neuronal cells are glial and endothelial cells, and in theory should be modeled as two separate compartments. However, because vasculature consists of only about 2–4% of the volume of the parenchyma (Lawers et al., 2008; Karbowski, 2011), it is safe to consider that the vast majority of non-neuronal cells are glial cells. Thus, for the sake of simplicity, here we model the brain parenchyma as consisting of only two compartments: glial (approximated as the number of non-neuronal cells) and neuronal. For the sake of simplicity, we heretofore refer to non-neuronal cells as “glial” cells, with the understanding that our model ignores an estimated 2–4% of the volume of the brain (that occupied by the vasculature).

Least-squares regressions to linear and power functions were calculated with PASW18 (IBM, USA). Mathematical models were developed with Mathematica (Wolfram Research, USA), and are fully described in the Results.

## Results

### Different neuronal scaling rules, similar non-neuronal scaling rules

For each of the brain structures analyzed of a particular species, let

***M*** be the total structure mass,

***N_n_*** be the total number of neurons in the structure,

***N_g_*** be the total number of glial cells in the structure,

***m_n_*** be the average mass of individual neuronal cells in the structure, including all its axonal and dendritic arbors, and

***m_g_*** be the average mass of individual glial cells in the structure, such that

***M_n_*** is the total neuronal mass in the structure, and

***M_g_*** is the total glial mass in the structure.

The mass of any brain structure (excluding the vascular component, which for the present purposes is considered negligible) can be divided in a neuronal component with mass ***M_n_*** = ***m_n_. N_n_*** and a glial component with mass ***M_g_*** = ***m_g_. N_g_***. Because ***m_n_*** and ***m_g_*** include the entire cell plus its pericellular space, no brain mass is left unaccounted for, as all tissue either belongs to ***M_n_*** or to ***M_g_***.

Empirically, in the 27 species analyzed here, we have found that the mass of a given brain structure (cerebral cortex, cerebellum or the remaining areas) for each order can be described to vary as power functions of ***N_n_*** and ***N_g_*** in the structure, such that

(1.1) $$M=k_{n}N_{n}^{\alpha n}$$

and

(1.2) $$M=k_{g}N_{g}^{\alpha g}$$

**Figure 1:**
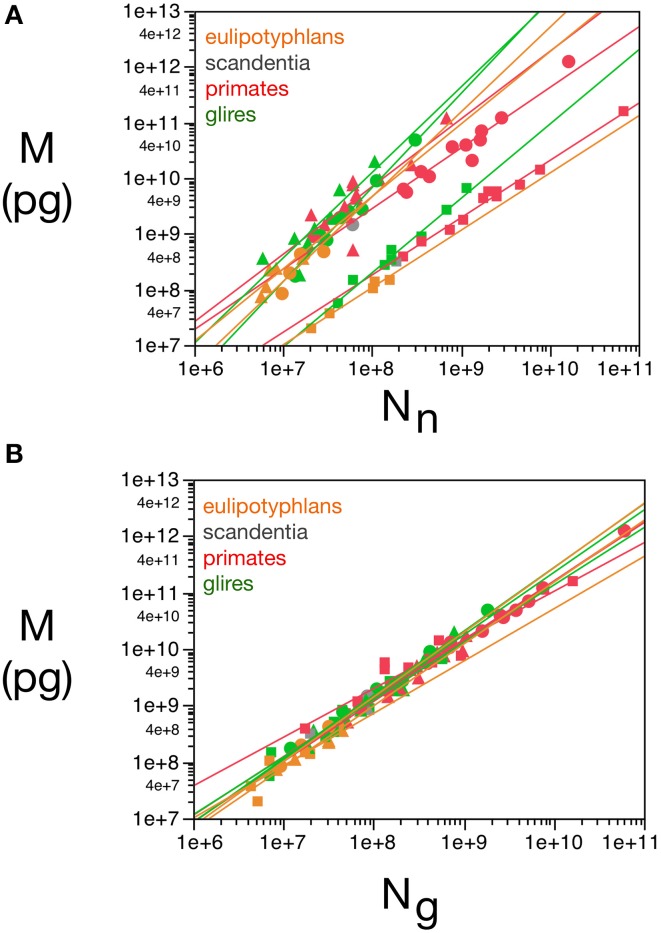
**Variation in structure mass as a function of number of neurons and glial cells in the structure**. Average brain structure mass for each species is plotted as a function of its total number of neurons **(A)** and non-neuronal (glial) cells **(B)**. Structure mass is given in picograms. Power functions are plotted separately for cerebral cortex (circles), cerebellum (squares) and rest of brain (triangles) in eulipotyphlans (orange), primates (red) and rodents (green). Power function constants and exponents are listed in Table 1. The two graphs are plotted with identical scales for comparison. Notice that the power functions are overlapping in **(B)**, but not in **(A)**. Data from Herculano-Houzel et al. (2006, 2007, 2011), Azevedo et al. (2009), Sarko et al. (2009), and Gabi et al. (2010).

These relationships are illustrated in Figure 1, and all constants and exponents are listed in Table 1. While different neuronal scaling exponents apply to different structures and mammalian orders (Figure 1A), similar glial scaling exponents apply across structures, species, and orders (Figure 1B). Indeed, the comparison of the values of ***k_n_***, ***k_g_***, **α_*n*_**, and ***α_g_*** for each structure and mammalian order shows that while ***α_n_*** and ***k_n_*** are highly variable, ***α_g_*** and ***k_g_*** vary little across all cases considered (Table 1), to such an extent that it is visually hard to distinguish between points corresponding to numbers of glial cells of different orders and structures.

**Table 1:** **Constants and exponents for neuronal and non-neuronal scaling rules**.

	**Neuronal scaling rules**		**Glia cells scaling rules**	
	***k_n_ (g)***	***α_n_***	***k_g_ (g)***	***α_g_***
**CEREBRAL CORTEX**				
Rodents	2.093 × 10^−13^	1.688	1.439 × 10^−9^	1.128
Primates	5.674 × 10^−9^	1.087	6.941 × 10^−9^	1.036
Eulipotyphlans	8.060 × 10^−13^	1.598	1.015 × 10^−9^	1.143
**CEREBELLUM**				
Rodents	4.172 × 10^−12^	1.334	9.267 × 10^−9^	1.016
Primates	1.084 × 10^−9^	1.028	2.564 × 10^−7^	0.861
Eulipotyphlans	1.946 × 10^−10^	1.297	1.726 × 10^−9^	1.094
**REST OF BRAIN**				
Rodents	6.711 × 10^−12^	1.534	1.985 × 10^−9^	1.105
Primates	1.084 × 10^−9^	1.214	3.512 × 10^−9^	1.065
Eulipotyphlans	1.946 × 10^−10^	1.297	2.819 × 10^−8^	0.926

*Constants **k_n_** and **k_g_** and exponents **a_n_** and **a_g_** relate structure mass to the number of neurons or other cells that compose the structure according to Equations 1.1 and 1.2, which are respectively **M = k_n_ N _n_^αn^** and **M = k_g_ N _g_^αg^***.

### Scaling of average neuronal and other cell mass

The existence of power laws relating the size of brain structures to numbers of neuronal and glial cells is hardly surprising. Indeed, the fact that primate, eulipotyphlan and rodent brains vary in size and cell numbers by five orders of magnitude while maintaining recognizable common overall macroscopic architectures implies that there must be general quantitative rules, or scale-invariant laws, relating the various measurable quantities that remain valid over the entire range of brain sizes. The only scale-invariant relation between two variables is a power law of the form ***y* = *c x^a^***. Of course, other more complicated scale-invariant laws relating more than two variables at a time are possible. But, for the sake of simplicity, and given the fact that the quantities that we can measure are always related by power laws, we will assume that the quantities that we cannot measure directly are also expressed as power law relations with respect to some structure size parameter (in this case, cell numbers). We have previously shown (Herculano-Houzel et al., 2006) that, assuming that average neuronal and glial cell masses are also related to the total structure mass by power laws with exponents ***β_n_*** and ***β_g_***, then it results that ***β_n_* = *α_n_ - 1*** and ***β_g_* = *α_g_ - 1***. Empirically, we find that ***β_g_* ≈ *0*** for rodents and primates, for all structures considered, while ***β_n_*** is variable across structures and orders (Table 1). This suggests that while the average neuronal mass differs across structures, species and orders, the average glial cell mass is approximately invariant for each structure and order. This analysis, of course, makes no use of the information contained in the residues of the power law best fit.

### Constant neuronal and non-neuronal mass fractions per structure and order

Given that, by our definition, the mass of any brain structure can be considered to be composed of a neuronal component ***M_n_* = *m_n_N_n_*** plus a glial component ***M_g_* = *m_g_N_g_***, then the mass of any brain structure can be said to consist of two fractions: a neuronal fraction ***f_n_* = *M_n_/M***, and a glial fraction ***f_g_* = *M*_*g*_/*M***.

When data from all structures and species are pooled, we find that the variation in structure mass spanning over four orders of magnitude, expressed as a function of the number of glial cells (also spanning fours orders of magnitude), is well-fitted by a power law that is approximately linear: ***M* = *k*_g_*N***_g_**^αg^**, with ***α_g_*** = 1.078 ± 0.170 (*p* < 0.0001) and ***k_g_*** = 1.673 ± 0.030 ng (*p* < 0.0001; Figure 1B). The common constant and the shared power law exponent suggest that characteristics related to glial cell size are shared across species, orders, and structures.

### Correlated scaling of cell densities across structures and orders

As explained above, the inverse of neuronal density does not amount to average neuronal cell mass because the relationship between neuronal cell density and average neuronal cell size depends on the fraction of tissue composed by neurons. This relationship can be defined mathematically as follows. The average neuronal and glial cell mass can be shown to be inversely proportional to the measured neuronal and glial cell densities ***d_nmes_*** and ***d_gmes_***, expressed in numbers of cells per unit of structure mass (here in picograms), and directly proportional to the mass fractions ***f_n_*** and ***f_g_***. This follows from writing cell densities as functions of mass fractions:

(2.1) $$d_{nmes}^{-1}=M/N_{n}=M_{n}/(N_{n}.f_{n})=m_{n}/f_{n}$$

(2.2) $$d_{gmes}^{-1}=M/N_{g}=M_{g}/(N_{g}.f_{g})=m_{g}/f_{g}$$

Note that the equations above express quantities that can be measured directly (the densities) as simple functions of quantities that cannot (the average cell type masses and total mass fractions). We next estimate the latter from the relationship between the quantities *d_nmes_* and *d_gmes_*.

We use the inverses of the densities instead of the densities themselves because these inverse quantities have a more natural interpretation, which is the average structure mass per unit cell of each type. The quantities ***d^−1^_nmes_*** and ***d^−1^_nmes_*** are upper bounds for the average mass of respectively neuronal and glial cells; more specifically, ***d^−1^_nmes_*** and ***d^−1^_nmes_*** should be proportional to the cube of the mean distance between neuronal cell bodies, and between glial cell bodies, respectively. If the average glial cell mass and glial mass fraction were to be completely invariant, then ***d^−1^_gmes_*** would be constant, and ***d^−1^_nmes_*** would increase proportionally to ***m_n_***; thus, the ratio between the average neuronal mass of each structure in each species would simply be proportional to the respective ***d^−1^_nmes_*** ratio. We consider this a zeroth-order version of our model, from which we could conclude, based on our previously published experimental data, that, for instance, neurons in rodent brains increase in mass significantly as structure size increases, while average neuron mass in primate brain structures remain approximately constant. This conclusion was supported recently by the experimental findings of Elston and Manger (2014), and is reviewed in Herculano-Houzel et al. (2014a).

This zeroth-order model can be made more precise by the introduction of extra terms that better take into account the empirical relationship between ***d^−1^_nmes_*** and ***d^−1^_gmes_***, and which can provide estimates of the average cell masses themselves.

**Figure 2:**
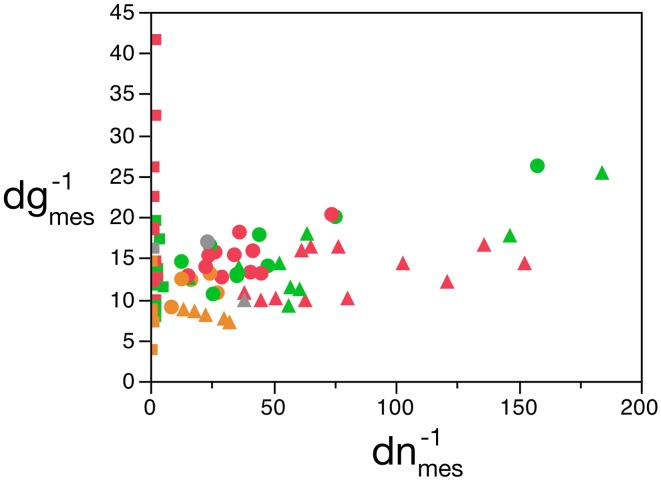
**Covariation in glial and neuronal density across brain structures and species**. The inverse of neuronal (X-axis) and glial (Y-axis) cell density, expressed as picograms per cell, is plotted for each species and brain structure: cerebral cortex (circles), cerebellum (squares) and rest of brain (triangles). Data from Herculano-Houzel et al. (2006, 2007, 2011), Azevedo et al. (2009), Sarko et al. (2009), and Gabi et al. (2010).

We find that, in the cerebellum, ***d^−1^_gmes_*** has a large relative variance (the largest for any structure), with values that are not correlated with ***d^−1^_nmes_*** (Pearson's correlation *R* = 0.15, with *p* = 0.47 for the uncorrelated null hypothesis with this sample size). If we exclude the cerebellum from our analysis (considering that the enormous disparity in size between granular and Purkinje neurons therein makes using average neuronal mass as a variable somewhat problematic), we find that ***d^−1^_nmes_*** varies greatly (by a factor of over 100-fold, and a coefficient of variation of 0.80), while ***d^−1^_gmes_*** varies more modestly (3.6 times, coefficient of variation = 0.29) but is positively correlated with ***d^−1^_nmes_*** (*R* = 0.58, *p* = 2 × 10^−6^; Figure 2). Therefore, at least for non-cerebellar structures, neuronal and glial cell densities are correlated.

### Modeling of *f*_*G*_ and *m*_*G*_

From the small variation in the relationship M × N_*g*_ (Figure 1B), and thus in d_*g*_, we expect the variability of both ***f_g_*** and ***m_g_*** to be small (since ***d*^−*1*^_*gmes*_ = *m*_*g*_/*f*_*g*_**). More precisely, we expect each of these variables to be expressed as slowly varying functions of the correlated variables (***m_n_*, *d^−1^_nmes_*** or ***d^−1^_gmes_***). But these latter variables are not independent of each other (remember that *f*_*g*_ = 1 − *f*_*n*_), so in each case we can always express two of them in terms of the third. Thus, in the spirit of parsimony, we expect that the values of ***f_g_*** and ***m_g_*** should be well-approximated in each case by a first-order Taylor series expansion on a single coordinate variable: A constant term, plus a small linear correction proportional to said variable. If there were exactly no variability, and up to first order, the choice of coordinate variables would not matter. The question then becomes finding the choice of two pairs of dependent and independent variables that minimizes discrepancy between theoretical and actual values of ***f_g_*** and ***m_g_***. In other words, we ask: What pairs of variables amongst *m_n_*, *m*, *f_n_*, *f_g_*, ***d^−1^_gmes_***, and ***d^−1^_nmes_*** are more strongly correlated?

To answer this question, we note that ***f_g_*** and ***d^−1^_gmes_*** are both slowly varying global properties of glial cells, measured for the entire brain structure. We should therefore expect these two variables to be more strongly correlated with each other than with ***m_n_*** or ***d^−1^_nmes_***. Thus, our model's discrepancy should be minimized by writing

(3) $$fg=fg_{0}+u\;d_{gmes}^{-1}$$

Glial cells interact with and support neurons in various ways. Unlike neurons, their action is more localized to neighboring cells. It therefore stands to reason that if any external factor influences the fully-developed size of a glial cell, it should be either intrinsic (genetic, for instance) or a factor related to the local conditions of its neighboring neurons, such as the average neuron size in its neighborhood, which determines the size of interstitial spaces between closely packed neurons. Therefore, and given the correlation between ***d_nmes_*** and ***d_gmes_***, we postulate that ***m_g_*** depends on ***m_n_***, such that it is well approximated by

(4) $$m_{g}=mg_{0}+v.m_{n}.$$

The combination of the equations above is the simplest possible away of modeling the relation between neuronal and glial cell densities, in a way that predicts univocally as functions of ***d_gmes_*** and ***d_nmes_*** the values of ***m_g_***, ***m_n_***, ***f_g_***, and ***f_n_***. Because these are not independent variables, and given the empirical values of the densities, the determination of any one of them is sufficient to allow the determination of the other three.

We can now obtain the model-dependent expected value ***d^−1^_g_*** as an implicit function of ***d*^−1^_*n*_** by combining Equations 2, 3, and 4 and substituting ***d^−1^_nmes_*** by ***d^−1^_g_*** whenever it occurs:

(5) $$\begin{array}{l} d_{g}^{-1}=m_{g}/f{\text{​}}_{g}=[m_{g0}+v.d_{n}^{-1}.(1-f{\text{​}}_{g0}-u.d_{g}^{-1})]\\ \;/(f{\text{​}}_{g0}+u.d_{g}^{-1}) \end{array}$$

The equation above can be solved for ***d^−1^_g_***, resulting in an expression of ***d^−1^_g_*** as a function of ***d^−1^_n_*** and four adjustable parameters ***f_g0_***, ***m_g0_***, ***u***, and ***v***. Thus, for each choice of said parameters one would obtain a different curve ***d^−1^_g_*** (***d^−1^_n_***). Following the standard recipe for chi-square minimization (Chernoff and Lehmann, 1954), for each such curve we then compute the value of the chi-square function χ^2^ = ∑ **(*d_g_*^−1^** – ***d_gmes_*^−1^)**^2^/**σ^2^*_dgmes^−1^_*** by comparison with the actual data points, and choose the combination of coefficients that minimizes its value. Since the standard deviation of the function ***d^−1^_g_*** – ***d***_**g**_^**−1**^_**mes**_ cannot be measured, we assume that all variations of ***d^−1^_g_*** are unbiased Gaussian fluctuations around ***d^−1^_gmes_***, so that the total χ^2^ is equal to the number of degrees of freedom.

**Figure 3:**
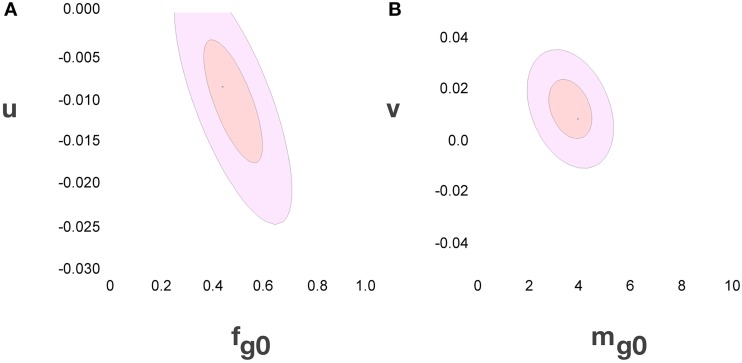
**Uncertainty ellipses for parameters in the model**. **(A)** Estimates for u and ***fg_0_***. **(B)** Estimates for *v* and ***mg_0_***. The values that minimize the χ^2^ function are *u* = −0.010 ± 0.005 pg^−1^, *v* = 0.017 ± 0.009, ***f_g0_*** = 0.48 ± 0.08 and ***m_g0_*** = 3.6 ± 0.8 pg.

**Figure 4:**
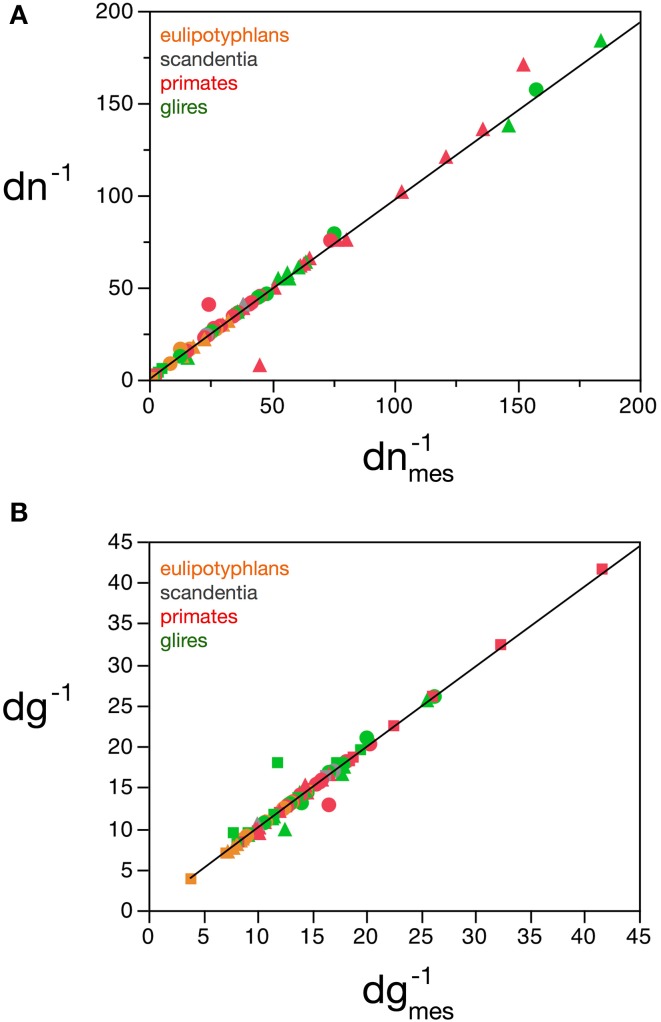
**Correspondence between measured and estimated cell densities**. **(A)** Graphs show the correspondence between measured and estimated values for the inverse of neuronal density estimated by the model, ***d_n_*^−1^**, and the inverse of measured neuronal density, ***d^−1^_nmes_***. **(B)** Correspondence between measured and estimated values for the inverse of glial density estimated by the model, ***d^−1^_g_***, and the inverse of measured glial density, ***d^−1^_gmes_***. Values expressed as picograms per cell, plotted for each species and brain structure: cerebral cortex (circles), cerebellum (squares) and rest of brain (triangles). Power fits plotted have exponents 0.991 ± 0.015, *r*^2^ = 0.983, *p* < 0.0001 **(A)** and 0.987 ± 0.021, *r*^2^ = 0.967, *p* < 0.0001 **(B)**. Data from Herculano-Houzel et al. (2006, 2007, 2011), Azevedo et al. (2009), Sarko et al. (2009), and Gabi et al. (2010).

Considering the data points for the cortex and rest of the brain of all species analyzed, and using reasonable priors (***m_g0_*** > 0, ***f_g0_*** > 0, ***k*** < 0, ***f_g_*** < 0.75, and |***v***| < 0.05), the values that minimize the χ^2^ function and provide the best value of ***d^−1^_g_*** that fits ***d^−1^_gmes_*** are ***f_g0_*** = 0.48 ± 0.08, ***m_g0_*** = 3.6 ± 0.8 pg, ***u*** = − 0.010 ± 0.005 pg^−1^ and ***v*** = 0.017 ± 0.009. The uncertainty ellipses described by the covariance matrix associated with these parameters are shown in Figure 3. Applying these values, we find a very good fit between the expected and measured values ***d^−1^_nmes_*** and ***d^−1^_n_***, and between ***d^−1^_gmes_*** and ***d^−1^_g_*** (power fit for ***d^−1^_n_*** × ***d^−1^_nmes_***, exponent 0.991 ± 0.015, *r*^2^ = 0.983, *p* < 0.0001; power fit for ***d^−1^_g_*** × ***d^−1^_gmes_***, exponent 0.987 ± 0.021, *r*^2^ = 0.967, *p* < 0.0001; Figure 4). The residuals of the relationship ***d^−1^_gmes_*** × ***d^−1^_nmes_*** are Gaussianly distributed with zero mean in comparison to the predicted ***d^−1^_g_*** × ***d^−1^_nmes_*** (Anderson-Darling test statistic *A*^2^ = 0.30, which is also better than the *A*^2^ = 0.37 obtained for a naïve linear fit; Shorack and Wellner, 1986).

### Values of *f*_*G*_, *m*_*G*_ and *m*_*N*_

**Figure 5:**
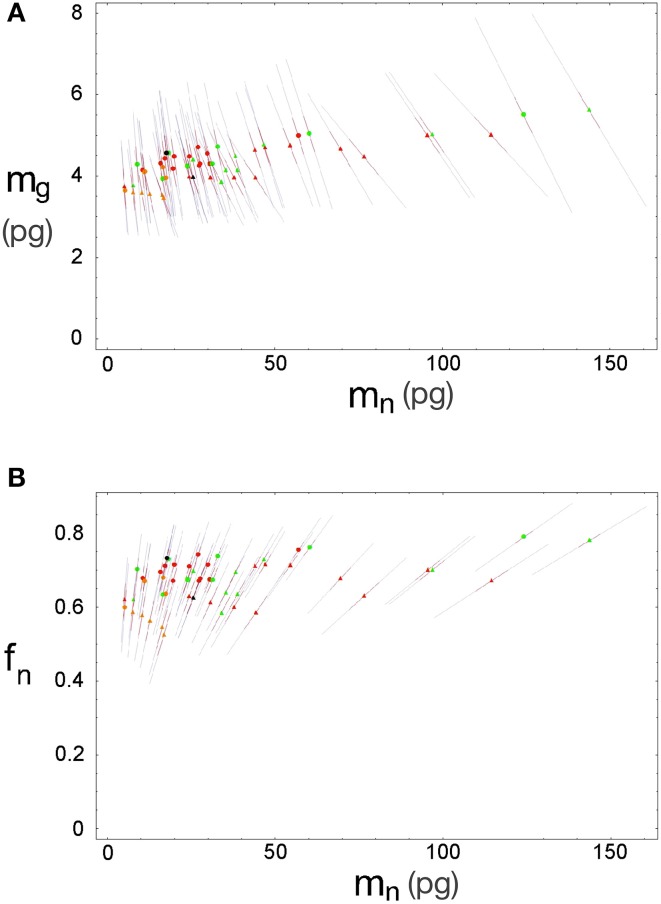
**Errors for estimated neuronal and glial cell masses and neuronal mass fraction**. Each point indicates the estimated average neuronal cell mass, ***m_n_***, and average glial cell mass, ***m_g_*** **(A)** or neuronal mass fraction, ***f_n_*** **(B)** for a given brain structure in a species: cerebral cortex (circles), cerebellum (squares) and rest of brain (triangles) in eulipotyphlans (orange), primates (red) and rodents (green). Errors are shown as bars ranging from each point.

Inserting the values of ***f_g0_***, ***m_g0_***, ***u*** and ***v*** obtained by χ^2^ minimization into Equations 3 and 4 gives us the expected values of ***f***_***g***_ and ***m***_***g***_. The discrepancy between the actual and expected values of ***d^−1^_g_*** given by Equation 5 arises from the unknown discrepancies between the actual and expected values ***f***_***g***_ and ***m***_***g***_. Assuming theses discrepancies are uncorrelated and Gaussianly distributed, their greatest joint likelihood occurs for δ ***f***_***g***_/***f***_***g***_ = −δ ***m***_***g***_/***m***_***g***_ when Equation 2.2 is satisfied. We can thus obtain the best-fit values for ***f***_***g***_ and ***m***_***g***_, (and thus ***f***_***n***_ and ***m***_***n***_), along with two complementary error estimates: the uncorrelated error given by the variance of δ ***f***_***g***_ (or, equivalently, δ ***m***_***g***_) due to inter-species and -structure variability, and the correlated modeling error due to the uncertainties in the parameters ***f_g0_***, ***m_g0_***, ***u***, and ***v*** given by their corresponding covariance matrix (given visual expression in the error ellipses of Figure 3). In short, the former error affects each data point separately, and the latter error affects all of them together. Also, since the determination of any of the variables ***f***_***g***_, ***m***_***g***_, ***f***_***n***_, or ***m***_***n***_ is sufficient (along with knowledge of ***d^−1^_gmes_*** and ***d^−1^_nmes_***) to determine all other variables, their respective errors will be fully degenerate, and we find that the uncorrelated and (much larger) correlated errors will take the form of collinear inclined error bars around each point (Figure 5).

**Figure 6:**
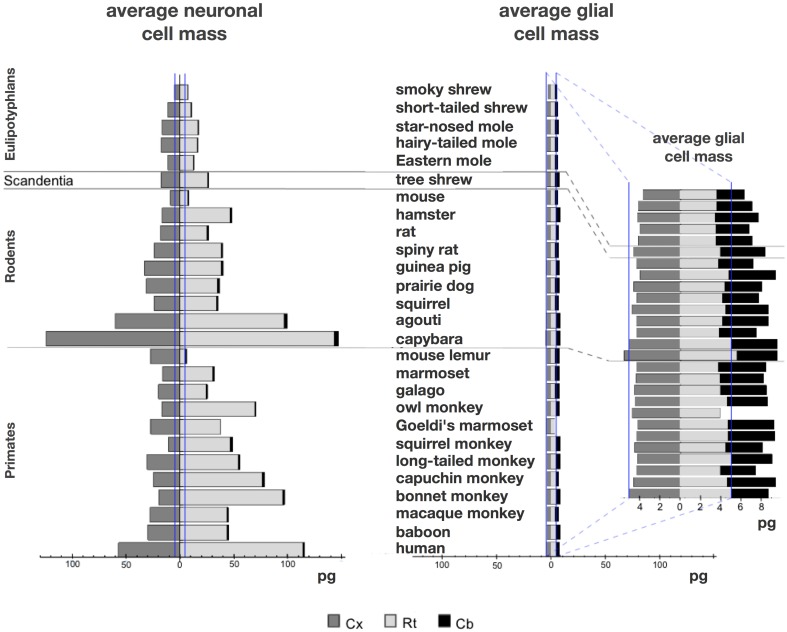
**Large variation in average neuronal cell mass, but not in average glial cell mass**. Main figure, horizontal bars (stacked, non-overlapping) indicate estimated average neuronal (left) and glial (right) cell mass in the cerebral cortex (dark gray), cerebellum (black) and rest of brain (light gray) for each species. Neuronal and glial cell masses are shown on same scale for comparison. Inset, estimated average glial cell masses shown on a larger scale. Values per structure and species are given in **Table 5**.

**Figure 7:**
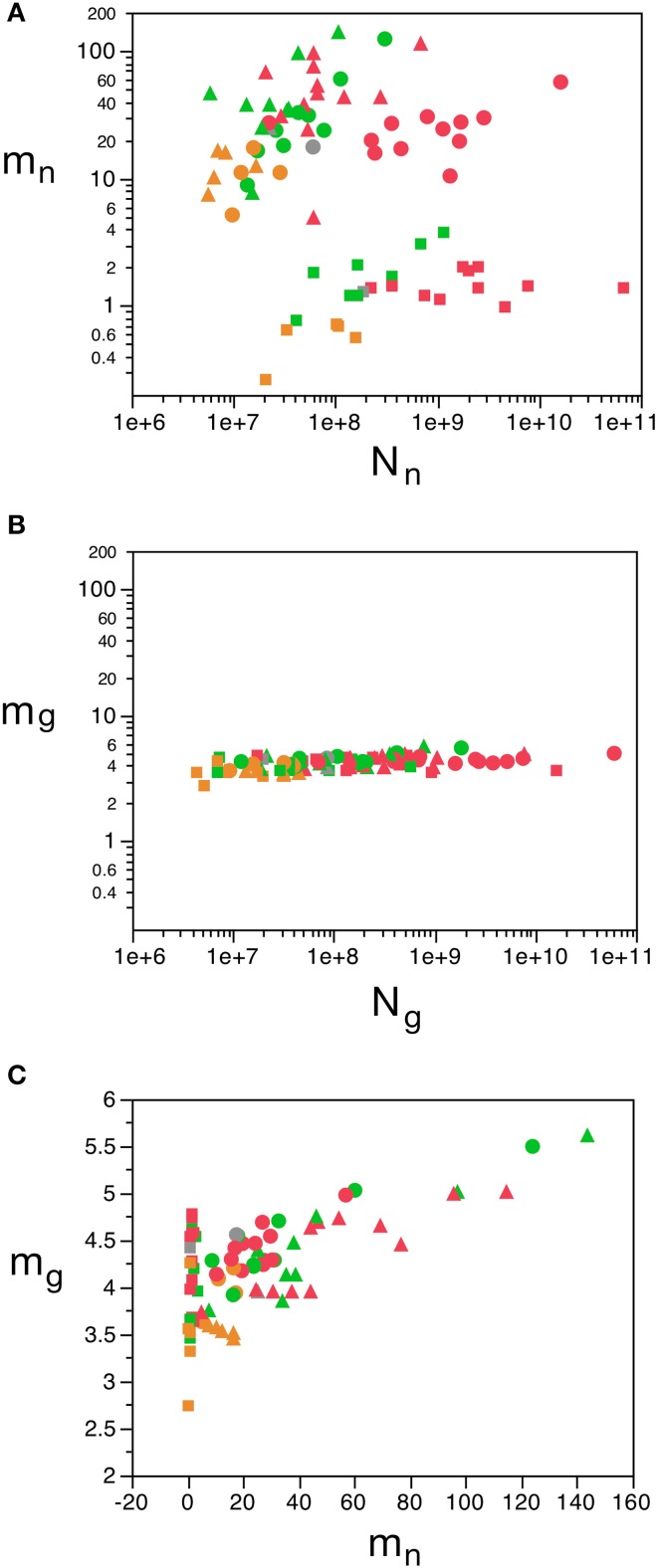
**Variation in predicted average neuronal and glial cell mass as a function of number of neurons and glial cells in the structure. (A)** Estimated average neuronal cell mass (*m_n_*) for each brain structure in each species is plotted as a function of the total number of neurons in the structure (*N_n_*). **(B)** Estimated average glial cell mass (*m_g_*) for each brain structure in each species is plotted as a function of the total number of glial cells in the structure (*N_g_*). **(C)** Estimated average glial cell mass plotted as a function of estimated average neuronal cell mass in each brain structure and species. Cell mass given in picograms. Cerebral cortex plotted as circles, cerebellum as squares, and rest of brain as triangles; eulipotyphlans shown in orange, primates in red, and rodents in green. Graphs in **(A,B)** are plotted with identical scales for comparison, to illustrate the large variation in *m_n_* but small variation in *m_g_*. Data from Herculano-Houzel et al. (2006); Herculano-Houzel et al. (2007); Herculano-Houzel et al. (2011), Azevedo et al. (2009), Sarko et al. (2009), and Gabi et al. (2010).

We find that the predicted values of ***m_n_*** are highly variable, ranging between 5 pg and 144 pg across all orders across the cerebral cortex and rest of brain (or between 0.26 and 144 pg if we apply the model to the cerebellum), with a variation of 29-fold (or 544-fold including the cerebellum, as expected given the large number of small granule cell neurons therein; Tables 2, **5**, Figure 6). The relationship between average neuronal mass and structure mass or number of neurons is homogeneous neither across structures nor across mammalian orders (Figure 7A). Average neuronal cell mass in a structure increases coordinately with number of neurons (and structure mass) in the rodent and eulipotyphlan cerebral cortex, in the rodent cerebellum, and in rodent and insectivore remaining brain areas, while no correlation is found for any structure across primate species (Figure 7A). Notice that values of ***m_n_*** (as well as of ***m_g_***) for the cerebral cortex, cerebellum, and rest of brain apply to the ensemble of gray and white matter in each structure, and thus include the neuronal mass distributed across both portions.

**Table 2:** **Predicted average neuronal cell mass in mammalian brain structures**.

	**Mean mass ± *SD* (pg)**	**Minimum**	**Maximum**	**Variation**
**CEREBRAL CORTEX**				
All orders	26.829 ± 23.130	5.163	124.051	24.0×
Eulipotyphlans	12.263 ± 4.939	5.163	17.363	3.4×
Rodents	37.697 ± 35.502	8.863	124.051	14.0×
Primates	25.513 ± 11.676	10.438	56.916	5.5×
Scandentia	17.629			
**REST OF BRAIN**				
All orders	44.372 ± 34.786	5.004	143.644	28.7×
Eulipotyphlans	12.707 ± 3.925	7.588	16.783	2.2×
Rodents	51.776 ± 41.959	7.693	143.644	18.7×
Primates	53.582 ± 30.800	5.004	114.364	22.9×
Scandentia	25.522			
Cortex + RoB, all orders	35.600 ± 30.569	5.004	143.644	28.7×
**CEREBELLUM**				
All orders	1.440 ± 0.756	0.264	3.775	14.3×
Eulipotyphlans	0.578 ± 0.183	0.264	0.714	2.7×
Rodents	1.889 ± 0.956	0.756	3.775	5.0×
Primates	1.479 ± 0.355	0.976	2.028	2.1×
Scandentia	1.283			
All structures, all orders	24.498 ± 29.771	0.264	143.644	544.1×

*Variation, maximum/minimum*.

In contrast to the large variation in ***m_n_***, the predicted values of ***m_g_*** vary by only 1.4-fold (between 3.45 and 5.62 pg) across the cerebral cortex and rest of brain across orders, around a mean of 4.31 ± 0.48 pg, which is larger than the average neuronal mass in the cerebellum but about 7 to 11-fold smaller than the average neuronal mass in the cerebral cortex and remaining structures (Table 3, Figures 5, 6). Despite the small range of values, variations in the predicted ***m_g_*** are significantly correlated with structure mass (and number of glial cells; Figure 7B) in rodent and insectivore cerebral cortex and in the rest of brain, where we have seen a significant variation in ***m_n_***, but in none of the primate structures nor in the cerebellum, where ***m_n_*** varies little. Corroborating the hypothesis in our model (Equation 4), we find that ***m_g_*** is a slowly-varying linear function of ***m_n_*** across the cerebral cortex and rest of brain (Figure 7C). There are, however, uncorrelated deviations of ***m_g_*** from this slowly-varying linear function of ***m_n_***. These deviations are typically small (a relative standard deviation of 7% for non-cerebellar glial cells, or 10% for all glial cells), but are determinant of the variation in ***f_g_***, as this is correlated only with ***m_g_*** and not with ***m_n_*** (see below).

**Table 3:** **Predicted average glia cell mass in mammalian brain structures**.

	**Mean mass ± *SD* (pg)**	**Minimum**	**Maximum**	**Variation**
**CEREBRAL CORTEX**				
All orders	4.380 ± 0.377	3.634	5.497	1.5×
Eulipotyphlans	3.994 ± 0.222	3.634	4.205	1.2×
Rodents	4.527 ± 0.486	3.919	5.497	1.4×
Primates	4.417 ± 0.239	4.139	4.980	1.2×
Scandentia	4.560			
**REST OF BRAIN**				
All orders	4.246 ± 0.567	3.454	5.619	1.6×
Eulipotyphlans	3.542 ± 0.059	3.454	3.600	1.0×
Rodents	4.463 ± 0.593	3.768	5.619	1.5×
Primates	4.400 ± 0.454	3.742	5.011	1.3×
Scandentia	3.964			
Cortex + RoB, all orders	4.313 ± 0.482	3.454	5.619	1.6×
**CEREBELLUM**				
All orders	4.017 ± 0.531	2.738	4.776	1.7×
Eulipotyphlans	3.476 ± 0.546	2.738	4.258	1.6×
Rodents	4.030 ± 0.458	3.462	4.628	1.3×
Primates	4.217 ± 0.464	3.495	4.776	1.4×
Scandentia	4.415			
All structures, all orders	4.217 ± 0.515	2.738	5.619	2.0×

*Variation, maximum/minimum*.

Like ***m_g_***, ***f_g_*** varies little across all structures and orders, around a mean of 0.33 ± 0.06 and between a minimum of 0.21 and a maximum of 0.48 in the cerebral cortex and rest of brain (Tables 4, 5), which indicates that its complement, the neuronal mass fraction of each brain structure, ***f_n_***, also varies little across brain structures and species (Table 4, Figure 5B). Notice that, as for ***m_n_*** and ***m_g_***, values of ***f_n_*** (as well as of ***f_g_***) for the cerebral cortex, cerebellum, and rest of brain apply to the ensemble of gray and white matter in each structure. An interesting way to illustrate the meaning of the neuronal mass fraction ***f_n_*** is to estimate the total neuronal mass in the various brain structures, which can be obtained as the product ***m_n_.N_n_***. For example, the human cerebral cortex, of total mass 1233 g (for the combined gray and white matter), with a neuronal mass fraction of 0.754 is estimated to consist of 930 g of neurons and 303 g of glial cells, and the mouse cerebral cortex, at 0.173 g total mass and a neuronal mass fraction of 0.701, is estimated to consist of 0.121 g of neurons and 0.052 g of glial cells (Table 5).

**Table 4:** **Predicted average glial mass fraction in mammalian brain structures**.

	**Mean fraction ± *SD* (pg)**	**Minimum**	**Maximum**	**Variation**
**CEREBRAL CORTEX**				
All orders	0.301 ± 0.042	0.211	0.402	1.9×
Eulipotyphlans	0.350 ± 0.034	0.320	0.401	1.3×
Rodents	0.293 ± 0.050	0.211	0.368	1.7×
Primates	0.300 ± 0.028	0.246	0.330	1.3×
Scandentia	0.268			
**REST OF BRAIN**				
All orders	0.357 ± 0.063	0.220	0.475	2.2×
Eulipotyphlans	0.441 ± 0.025	0.413	0.475	1.1×
Rodents	0.325 ± 0.061	0.220	0.416	1.9×
Primates	0.345 ± 0.048	0.284	0.415	1.5×
Scandentia	0.376			
Cortex + RoB, all orders	0.331 ± 0.059	0.211	0.475	2.3×
**CEREBELLUM**				
All orders	0.325 ± 0.125	0.087	0.724	8.3×
Eulipotyphlans	0.462 ± 0.160	0.293	0.724	2.5×
Rodents	0.332 ± 0.073	0.237	0.430	1.8×
Primates	0.261 ± 0.102	0.087	0.423	4.8×
Scandentia	0.271			
All structures, all orders	0.329 ± 0.086	0.087	0.724	8.3×

*Variation, maximum/minimum*.

**Table 5:** **Predicted average cell masses and neuronal mass fraction per species**.

**Species**	**Cerebral cortex**			**Cerebellum**			**Rest of brain**		
	***m_**N**_***	***m_**G**_***	***f_**N**_***	***m_**N**_***	***m_**G**_***	***f_**N**_***	***m_**N**_***	***m_**G**_***	***f_**N**_***
**PRIMATES**									
*Microcebus murinus*	27.265	4.242	0.670	1.393	4.738	0.789	5.004	3.742	0.619
*Callithrix jacchus*	15.779	4.299	0.694	1.434	4.280	0.710	30.687	3.977	0.629
*Otolemur garnettii*	19.877	4.466	0.714	1.206	4.543	0.749	69.455	4.658	0.678
*Aotus trivirgatus*	17.078	4.418	0.711	1.109	3.982	0.666	37.746	3.961	0.600
*Callimico goeldii*	26.961	4.692	0.742	n.a.	n.a.	n.a.	24.335	3.977	0.629
*Saimiri sciureus*	10.438	4.139	0.677	2.028	4.579	0.858	54.470	4.741	0.713
*Cebus apella*	24.373	4.468	0.709	1.395	4.580	0.755	95.391	5.000	0.700
*Macaca fascicularis*	30.450	4.290	0.673	2.001	3.649	0.912	46.947	4.694	0.716
*Macaca radiata*	19.549	4.176	0.670	1.914	4.072	0.679	76.478	4.470	0.630
*Macaca mulatta*	27.629	4.286	0.676	0.976	3.495	0.577	44.140	3.956	0.585
*Papio anubis cynocephalus*	29.847	4.544	0.714	1.441	4.776	0.817	43.972	4.634	0.710
*Homo sapiens*	56.912	4.980	0.754	1.374	3.689	0.616	114.364	5.001	0.671
**SCANDENTIA**									
*Tupaia glis*	17.629	4.560	0.732	1.283	4.414	0.730	25.522	3.964	0.624
**RODENTS**									
*Mus musculus*	8.863	4.284	0.701	0.756	3.463	0.570	7.692	3.768	0.619
*Mesocricetus auratus*	16.444	3.919	0.632	1.808	4.627	0.763	46.552	4.769	0.729
*Rattus norvegicus*	18.088	4.550	0.730	1.192	3.656	0.610	25.453	4.393	0.696
*Proechimys cayennensis*	23.792	4.222	0.672	1.216	3.610	0.601	38.656	4.135	0.633
*Cavia porcellus*	32.792	4.705	0.736	2.073	4.190	0.696	38.076	4.484	0.694
*Cynomys* sp.	31.248	4.290	0.672	1.680	4.545	0.749	35.215	4.133	0.639
*Sciurus carolinensis*	23.793	4.240	0.675	1.482	3.675	0.613	33.896	3.851	0.584
*Dasyprocta primnolopha*	60.200	5.031	0.761	3.019	4.544	0.747	96.799	5.015	0.700
*Hydrochoerus hydrochoeris*	124.051	5.497	0.789	3.775	3.962	0.659	143.644	5.619	0.780
**EULIPOTYPHLANS**									
*Sorex fumeus*	5.163	3.634	0.598	0.264	2.738	0.276	7.588	3.601	0.586
*Blarina brevicauda*	11.118	4.101	0.670	0.644	3.512	0.581	10.283	3.588	0.577
*Parascalops brewen*	16.566	4.205	0.679	0.714	4.258	0.707	16.783	3.454	0.525
*Condylura cristata*	17.363	3.941	0.634	0.693	3.312	0.532	16.300	3.520	0.545
*Scalopus aquaticus*	11.106	4.089	0.669	0.572	3.563	0.592	12.580	3.548	0.562

*Average neuronal and glial cell mass given in pg. Values apply to the ensemble of gray and white matter in each structure*.

**Figure 8:**
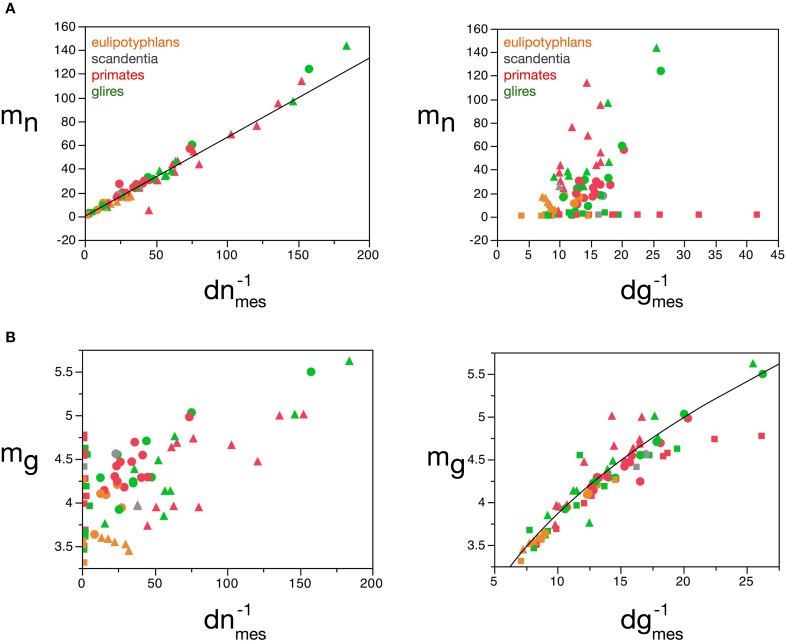
**Average neuronal and glial cell mass is well predicted by measured neuronal and glial cell densities, respectively**. **(A)** Estimated average neuronal cell mass (***m_n_***) for each brain structure in each species is plotted as a function of the inverse of the measured neuronal density in the structure (***d^−1^_nmes_***, left) and as a function of the inverse of the measured glial density in the structure (***d^−1^_gmes_***, right). **(B)** Estimated average glial cell mass (***m_g_***) for each brain structure in each species is plotted as a function of the inverse of the measured neuronal density in the structure (***d^−1^_nmes_***, left) and as a function of the inverse of the measured glial density in the structure (***d^−1^_gmes_***, right). Cell mass given in picograms, and the inverse of cell densities in picogram/neuron. Functions plotted are **(A)** ***m_n_*** = 0.649 **(*d^−1^_nmes_)***^1.004 ± 0.019^ (*r*^2^ = 0.973, *p* < 0.0001) and **(B)** ***m_g_*** = 1.648 **(*d^−1^_gmes_***)^0.370 ± 0.017^ (*r*^2^ = 0.897, *p* < 0.0001). Cerebral cortex plotted as circles, cerebellum as squares, and rest of brain as triangles; eulipotyphlans shown in orange, primates in red, and rodents in green. Data from Herculano-Houzel et al. (2006, 2007, 2011), Azevedo et al. (2009), Sarko et al. (2009). and Gabi et al. (2010).

Our model shows that ***m_n_*** can be estimated reliably for the cerebral cortex, cerebellum and rest of the brain from the measured neuronal cell density in the tissue, according to the function ***m_n_*** = 0.649 **(*d_nmes_*^−*1*^**)^1.004 ± 0.019^ (*r*^2^ = 0.973, *p* < 0.0001; Figure 8A), but not from measured glial cell density. Conversely, ***m_g_*** can be estimated reliably for the cerebral cortex and rest of the brain from the measured glial cell density in the tissue, according to the function ***m_g_*** = 1.648 **(*d_gmes_*^−1^**)^0.370 ± 0.017^ (*r*^2^ = 0.897, *p* < 0.0001; Figure 8B), but not from measured neuronal density.

**Figure 9:**
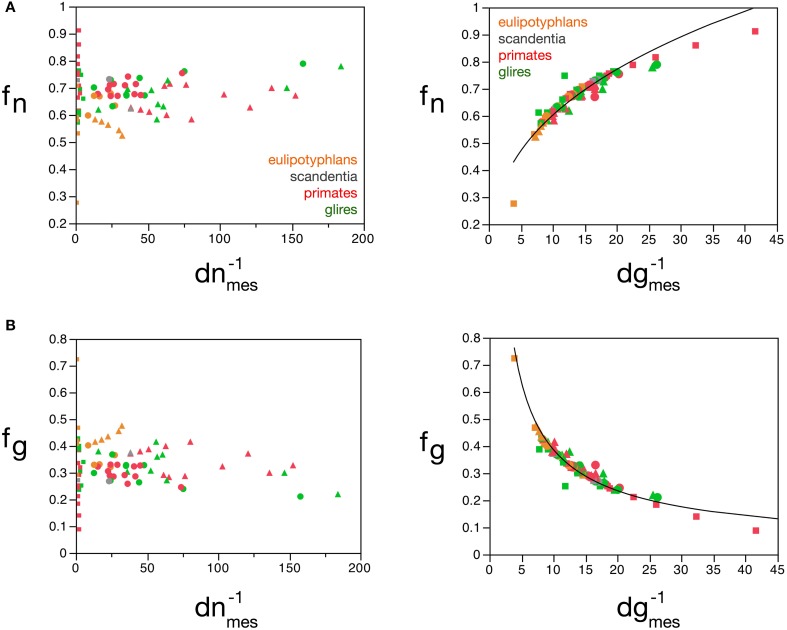
**Neuronal and glial mass fractions are well predicted by measured glial cell density**. **(A)** Estimated neuronal mass fraction (***f_n_***) in each brain structure in each species is plotted as a function of the inverse of the measured neuronal density in the structure (***d^−1^_nmes_***, left) and as a function of the inverse of the measured glial density in the structure (***d^−1^_gmes_***, right). **(B)** Estimated glial mass fraction (***f_g_***) in each brain structure in each species is plotted as a function of the inverse of the measured neuronal density in the structure (***d^−1^_nmes_***, left) and as a function of the inverse of the measured glial density in the structure (***d^−1^_gmes_***, right). Notice that neuronal (or glial) mass fraction is well predicted by variations in glial cell density, but not in neuronal cell density. Inverse of cell densities given in picograms/neuron. Functions plotted are **(A)** ***f_n_*** = 0.265 **(*d^−1^_gmes_***)^0.356 ± 0.019^ (*r*^2^ = 0.823, *p* < 0.0001) and **(B)** ***f_g_*** = 2.008 **(*d^−1^_gmes_***)^−0.716 ± 0.026^ (*r*^2^ = 0.906, *p* < 0.0001). Cerebral cortex plotted as circles, cerebellum as squares, and rest of brain as triangles; eulipotyphlans shown in orange, primates in red, and rodents in green. Data from Herculano-Houzel et al. (2006, 2007, 2011), Azevedo et al. (2009), Sarko et al. (2009), and Gabi et al. (2010).

The mass fractions ***f_g_*** and ***f_n_*** can also be estimated reliably from ***d_gmes_***, but interestingly not from ***d_nmes_*** (Figures 9A,B), for the cerebral cortex, cerebellum, and rest of brain, according to the equations ***f***_*n*_ = 0.265 **(*d^−1^_gmes_***)^0.356 ± 0.019^ (*r*^2^ = 0.823, *p* < 0.0001; Figure 9A) and ***f_g_*** = 2.008 **(*d^−1^_gmes_***)^−0.716 ± 0.026^ (*r*^2^ = 0.906, *p* < 0.0001; Figure 9B). Using cell densities obtained for the human brain (Azevedo et al., 2009), the human cerebral cortex is estimated to have an average neuronal cell mass (including components in both gray and white matter) of 48.37 pg, an average glial cell mass of 5.02 pg, and to have a neuronal mass fraction of 0.774—that is, 77.4% of the mass of the human cortex (both gray and white matter) is estimated to consist of neurons. In comparison, cell densities obtained for the mouse and capybara brains (Herculano-Houzel et al., 2006) allow us to estimate average neuronal cell masses in the mouse and capybara cerebral cortices of 8.33 and 104.53 pg, average glial cell masses of 4.44 and 5.52 pg, and neuronal mass fractions of 0.688 and 0.848, respectively. Notice that these values are very close to those estimated directly by the model (Table 5).

### Understanding diversity and determining universals

To investigate how the cellular composition of brain tissues varies and what parameters determine this variation, we used factor analysis of principal components of the variables ***M***, ***N****_n_*, ***N****_g_*, ***f****_g_*, ***m****_n_*, ***m****_g_*, ***N****_g_/****N****_n_*, ***m****_n_*.***N****_n_*, ***d****_n_*, and ***d****_g_*. This analysis yields a first component consisting of ***m****_g_*, ***m****_n_*, ***f****_g_* (or, equivalently, ***f****_n_*) and ***N****_g_/****N****_n_* that responds for 47.8% of variance across structures and species; a second component consisting of ***N****_n_*, ***N****_g_*, and ***m****_n_*.***N****_n_* that responds for an additional 27.8% of variance; and a third component, consisting of ***d****_n_* and ***d****_g_*, that accounts for another 9.0% of variance. This is compatible with the interpretation that the relationships between ***m****_g_*, ***m****_n_*, ***f****_g_* and ***N****_g_/****N****_n_* are universal across structures and species, while ***N****_n_* and ***m****_n_*.***N****_n_* are particular to given structures and species, and ***d****_n_* and ***d****_g_* are derived compound quantities.

**Figure 10:**
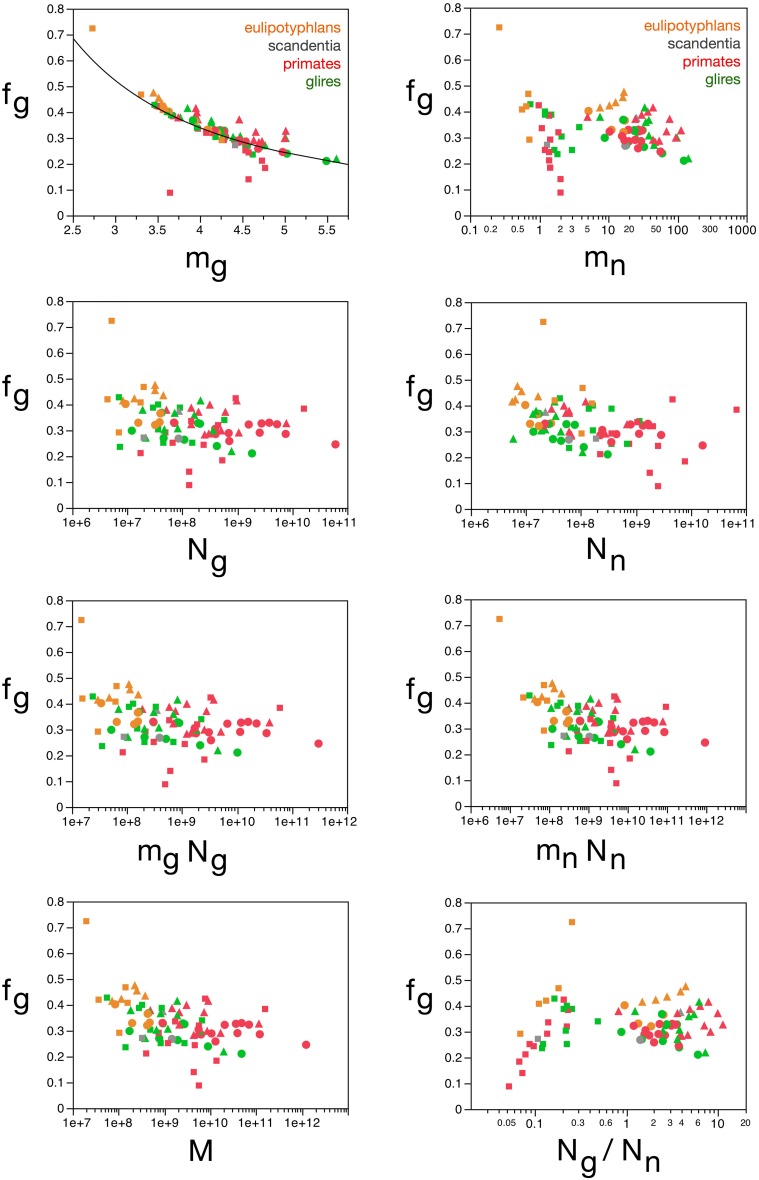
**Glial mass fraction varies with estimated average glial cell mass**. Graphs show the estimated glial mass fraction (***f_g_***) in each brain structure in each species plotted as a function of estimated average glial cell mass (***m_g_***), estimated average neuronal cell mass (***m_n_***), number of glial cells in the structure (***N_g_***), number of neurons in the structure (***N_n_***), total glial mass in the structure (***m_g_N_g_***), total neuronal mass in the structure (***m_n_N_n_***), total mass of the structure (M), and glia/neuron ratio in the structure (***N_g_/N_n_***). Notice that neuronal (or glial) mass fraction is only well predicted by variations in estimated average glial cell mass. All masses in picograms. Cerebral cortex plotted as circles, cerebellum as squares, and rest of brain as triangles; eulipotyphlans shown in orange, primates in red, and rodents in green. Data from Herculano-Houzel et al. (2006, 2007, 2011), Azevedo et al. (2009), Sarko et al. (2009), and Gabi et al. (2010).

Indeed, in agreement with the principal component analysis, we find that, while ***f****_g_* (and thus also ***f****_n_*) varies little across brain structures and species, around a mean of 0.33 ± 0.06, this variation in ***f****_g_* follows closely small variations in *m_g_*, while it is unrelated to other parameters (Figure 10). Considering that the glial mass fraction is a result of how the tissue is composed in development, this relationship indicates that small variations in average glial cell mass (and not in average neuronal cell mass) result in small variations in the glial mass fraction in the tissue (and thus also in the neuronal mass fraction).

**Figure 11:**
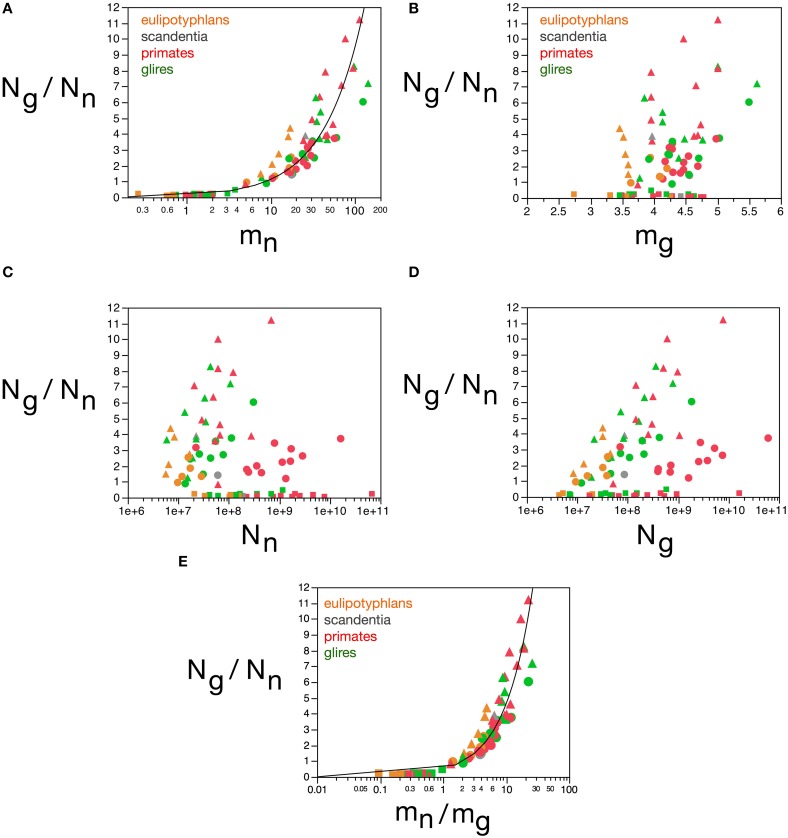
**Glia/neuron ratio is best predicted by average neuronal cell mass and by the ratio between average neuronal and glial cell masses**. Graphs show the glia/neuron ratio (*N_g_/N_n_*) in each brain structure in each species plotted **(A)** as a function of estimated average neuronal cell mass (*m_n_*), **(B)** as a function of estimated average glial cell mass (*m_g_*), **(C)** as a function of number of neurons in the structure (*N_n_*), **(D)** as a function of number of glial cells in the structure (*N_g_*), and **(E)** as a function of the ratio between average neuronal and average glial cell mass in the structure (*m_n_/m_g_*). Notice that glia/neuron ratio is only well predicted by variations in estimated average neuronal cell mass **(A)** and, even better, by the ratio between average neuronal and average glial cell mass in the structure. All masses in picograms. Functions plotted are **(A)** *N_g_/N_n_* = *0.137*(*m_n_*)^0.922 ± 0.035^ (*r*^2^ = 0.898, *p* < 0.0001) and **(E)** *N_g_/N_n_* = *0.487*(*m_n_/m_g_*)^0.977 ± 0.030^ (*r*^2^ = 0.929, *p* < 0.0001). Cerebral cortex plotted as circles, cerebellum as squares, and rest of brain as triangles; eulipotyphlans shown in orange, primates in red, and rodents in green. Data from Herculano-Houzel et al. (2006, 2007, 2011), Azevedo et al. (2009), Sarko et al. (2009), and Gabi et al. (2010).

The next parameter that loads in the first component is the ratio ***N****_g_/****N****_n_*. We had found previously that ***N****_g_/****N****_n_* varies similarly across brain structures and mammalian orders with the inverse of neuronal density (Herculano-Houzel, 2011a), which we considered to indicate that ***N****_g_/****N****_n_* increases with increasing average neuronal cell mass. This relationship is strikingly similar to the variation in glia/neuron ratios as a function of neuronal density for other species of carnivores, cetaceans, primates, and proboscideans (Herculano-Houzel, 2014). Indeed, here we find that the ***N****_g_/****N****_n_* ratio correlates strongly with the calculated ***m****_n_* (Spearman ρ = 0.9238, *p* < 0.0001), and varies as a power function of ***m****_n_* across all structures and species, such that ***N_g_/N_n_* = *0.137*(*m_n_***)^0.922 ± 0.035^ (*r*^2^ = 0.898, *p* < 0.0001; Figure 11A). In contrast, the ***N****_g_/****N****_n_* ratio varies less significantly with the calculated ***m****_g_* across structures and species (Spearman ρ = 0.2550, *p* = 0.0224; Figure 11B), with a large spread across brain structures and species. Similarly, and as shown before (Herculano-Houzel, 2011a), the ***N****_g_/****N****_n_* ratio is not correlated with either ***N****_g_* or ***N****_n_* across structures and species (Figures 11C,D). Considering that ***N****_g_/****N****_n_* ratio must result from factors that determine numbers of glial and neuronal cells in the tissue, these results indicate that the ***N****_g_/****N****_n_* ratio indeed depends on variations in ***m****_n_*, and not on ***m****_g_*, ***N****_g_* or ***N****_n_*. The glia/neuron ratio in any brain structure thus depends simply on the average size of the neurons in that structure.

**Figure 12:**
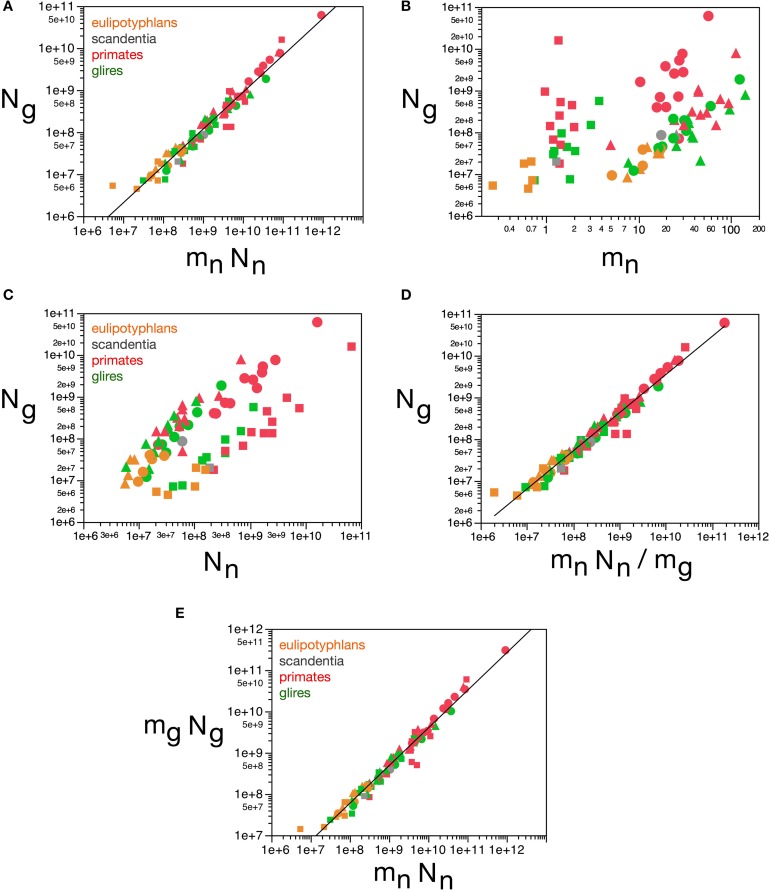
**Glial cell mass scales to match neuronal cell mass across brain structures and species**. Graphs show numbers of glial cells (***N_g_***) in each brain structure in each species plotted **(A)** as a function of total neuronal mass (***m_n_N_n_***), **(B)** as a function of estimated average neuronal cell mass (***m_n_***), **(C)** as a function of number of neurons in the structure (***N_n_***), **(D)** as a function of the neuronal mass per glial cell in the structure (***m_n_N_n_/m_g_***). **(E)** Total glial mass in each structure (***m_**g**_***N**_**g**_**) varies as a function of total neuronal mass in the structure (***m_n_N_n_***). Notice that numbers of glial cells in brain structures are well predicted by variations in total neuronal mass in the structure **(A)** and by the average neuronal mass per glial cell **(D)**. All masses in picograms. Functions plotted are **(A)** ***N****_**g**_* = **1.491 *m_n_***.***N***_*n*_^0.877 ± 0.022^ (*r*^2^ = 0.952, *p* < 0.0001), **(D) *m_n_*.*N_n_/m_g_***, with ***N****_g_* = **2.603 (*m_n_.N_n_/m_g_)***^0.913 ± 0.020^ (*r*^2^ = 0.965, *p* < 0.0001), and **(E)** ***M_g_*** = 3.078 ***M_n_***^0.911 ± 0.019^ (*r*^2^ = 0.968, *p* < 0.0001). Cerebral cortex plotted as circles, cerebellum as squares, and rest of brain as triangles; eulipotyphlans shown in orange, primates in red, and rodents in green. Data from Herculano-Houzel et al. (2006, 2007, 2011), Azevedo et al. (2009), Sarko et al. (2009), and Gabi et al. (2010).

This finding corroborates our previous model, which proposed that the ratio ***N****_g_/****N****_n_* is achieved as glial cells of nearly invariant size infiltrate the neuronal parenchyma (of total neuronal mass defined here as ***N****_n_*.***m****_n_*) and proliferate until achieving confluency (Herculano-Houzel et al., 2006; Herculano-Houzel, 2011a), resulting in a glia/neuron numeric ratio that depends solely on the average size of neurons in the tissue. Thus, the final number of glial cells in the tissue should be a function of the volume of the neuronal parenchyma to be infiltrated. Here we find that, indeed, ***N****_g_* varies across all structures and species as a single power function of the total neuronal mass of the structures (***m****_n_*.***N****_n_*) such that ***N****_g_* = **1.491 *m_n_***.***N_n_***^0.877 ± 0.022^ (*r*^2^ = 0.952, *p* < 0.0001; Figure 12A). In contrast, ***N_g_*** does not vary in a uniform manner across brain structures and species with variations in ***m_n_*** or in ***M_n_*** alone (Figures 12B,C). Considering that, in brain development, glial cells are only added in large numbers once numbers of neurons are already established, our findings attribute variations in ***N****_g_* to the product of variations in ***m****_n_* and in ***N****_n_*, that is, in total neuronal mass in the tissue.

**Figure 13:**
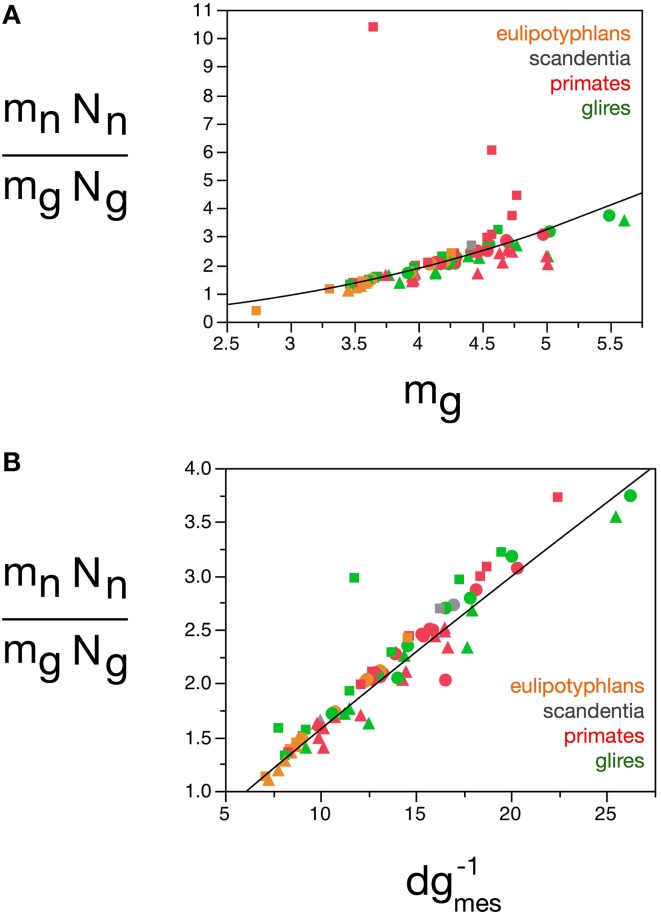
**Neuron/glia mass ratio varies with average glial cell mass across brain structures and species**. Graphs show the neuron/glia mass ratio (*m_n_N_n_/m_g_N_g_*) as **(A)** a function of estimated glial cell mass (***m_g_***) and **(B)** a function of the inverse of measured glial cell density (***d^−1^_gmes_***) in each brain structure in each species. Average glial cell mass in picograms; ***d^−1^_gmes_*** in picograms/neuron. Functions plotted are **(A)** ***m_n_.N_n_/m_g_.N_g_* = 0.064** ***m***_*g*_^2.433 ± 0.260^ (*r*^2^ = 0.528, *p* < 0.0001) and **(B)** ***m_n_.N_n_***/***m_g_.N_g_*** = **0.132 *d_gmes_*^−1^**^1.072 ± 0.034^ (*r*^2^ = 0.926, *p* < 0.0001). Cerebral cortex plotted as circles, cerebellum as squares, and rest of brain as triangles; eulipotyphlans shown in orange, primates in red, and rodents in green. Data from Herculano-Houzel et al. (2006, 2007, 2011), Azevedo et al. (2009), Sarko et al. (2009), and Gabi et al. (2010).

Here we find, however, that albeit varying little, as proposed in our previous model, glial cells do not have invariant mass; as shown above, variations in the average mass of glial cells in the tissue are consequent enough to actually impact on the glial mass fraction in the tissue. Once variations in ***m****_g_* are factored in, an even tighter relationship is found in how total glial cell mass, ***M_g_*** = ***m_g_.N_g_*,** varies together with total neuronal mass in the structures, ***M_n_*** = ***m_n_.N_n_*** in strictly the same fashion across all structures and species, in a way that can be described as ***M_g_*** = 3.078 ***M_n_***^0.911 ± 0.019^ (*r*^2^ = 0.968, *p* < 0.0001; Figure 12E). This implies that the total glial mass in a structure, ***M_g_*** = ***m_g_.N_g_***, is added in a similar way to all brain structures in all species that matches precisely the total neuronal mass in that structure at a certain ratio: To every certain amount neuronal mass corresponds a certain amount of glial mass in a predictable manner. This is supported by an even better relationship between ***N_g_*** and the ratio ***m_n_.N_n_/m_g_***, with ***N****_g_* = **2.603 (*m_n_.N_n_/m_g_***)^0.913 ± 0.020^ (*r*^2^ = 0.965, *p* < 0.0001; Figure 12D), which suggests that numbers of glial cells are added to match the total neuronal mass in the tissue, but in a way that depends on the precise average mass of the glial cells. Indeed, the ratio ***N_g_/N_n_***, which we found to vary as a function of ***m_n_***, is an even better function of the ratio ***m_n_/m_g_***, with ***N_g_/N_n_*** = **0.487(*m_n_/m_g_***)^0.977 ± 0.030^ (*r*^2^ = 0.929, *p* < 0.0001; Figure 11E). The ratio between total neuronal mass and total glial mass, ***m_n_.N_n_/m_g_.N_g_***, which is related (but not identical) to ***f****_n_*, varies between 1 and 3 as ***m****_g_* increases, such that ***m_n_.N_n_/m_g_.N_g_*** = **0.064 *m***_*g*_^2.433 ± 0.260^ (*r*^2^ = 0.528, *p* < 0.0001; Figure 13A), and even more tightly with measured glial cell density in the tissue, such that ***m_n_.N_n_/m_g_.N_g_*** = **0.132 *d*^−*1*^***_**gmes**_*^1.072 ± 0.034^ (*r*^2^ = 0.926, *p* < 0.0001; Figure 13B).

**Figure 14:**
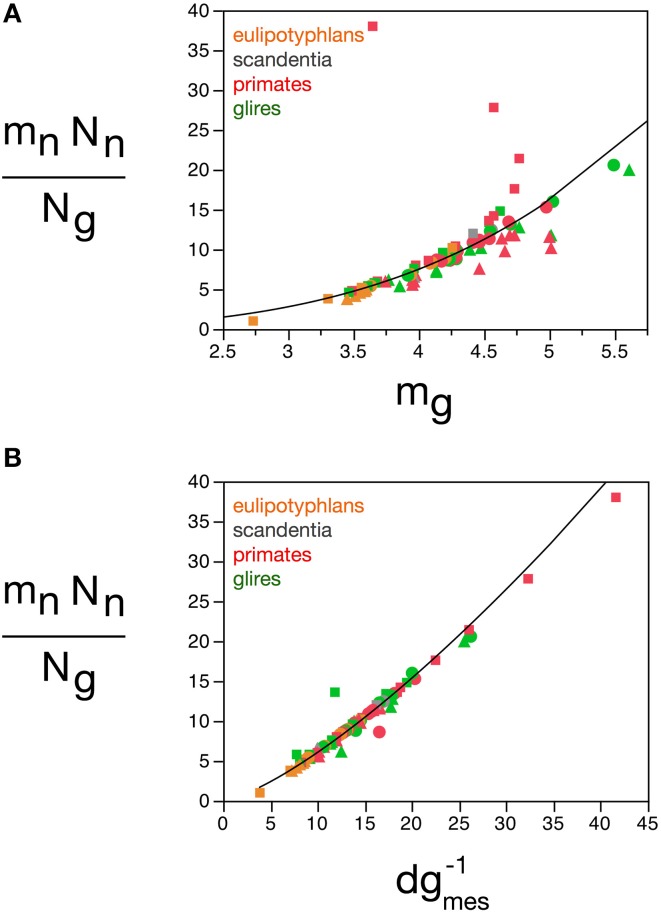
**Neuronal mass per glial cell varies with average glial cell mass across brain structures and species**. Graphs show the average neuronal mass per glial cell (*m_n_N_n_/N_g_*) as **(A)** a function of estimated glial cell mass (***m_g_***) and **(B)** a function of the inverse of measured glial cell density (***d^−1^_gmes_***) in each brain structure in each species. Average glial cell mass in picograms; ***d^−1^_gmes_*** in picograms/neuron. Functions plotted are (a) ***N_n_***.***m_n_***/***N_g_* = 0.064** ***m***_*g*_^3.433 ± 0.260^ (*r*^2^ = 0.691, *p* < 0.0001) and **(B)** ***m_n_.N_n_***/***N_g_*** = **0.275 *d*^−1^*_gmes_***^1.343 ± 0.033^ (*r*^2^ = 0.954, *p* < 0.0001). Cerebral cortex plotted as circles, cerebellum as squares, and rest of brain as triangles; eulipotyphlans shown in orange, primates in red, and rodents in green. Data from Herculano-Houzel et al. (2006, 2007, 2011), Azevedo et al. (2009), Sarko et al. (2009), and Gabi et al. (2010).

The quantitative match between the total glial mass in a structure and the total neuronal mass can be conceptualized as a fundamental building block of brain tissue across species and orders that corresponds to a certain amount of neuronal matter “assigned” to each glial cell (or vice-versa). This block is not invariant in mass, however, as indicated by the fact that the relationship between total glial mass and total neuronal mass is not linear, but rather a power function of exponent 0.911, slightly but significantly below 1.0. Calculating the ratio ***N_n_***.***m_n_***/***N_g_***, we find most values between 5 and 20 ng of neuronal mass per glial cell (Figure 14), which increase together with ***m_g_***: the larger the average glial cell, the larger the neuronal mass with which it is associated, in a mathematically predictable manner such that ***N_n_***.***m_n_***/***N_g_*** = **0.064 *m****_g_*^3.433 ± 0.260^ (*r*^2^ = 0.691, *p* < 0.0001; Figure 14A). Again, the correlation with measured glial cell density in the tissue is even better, such that ***m_n_.N_n_***/***N_g_*** = **0.275 *d***^−1^*_**gmes**_*^1.343 ± 0.0 33^ (*r*^2^ = 0.954, *p* < 0.0001; Figure 14B). Thus, the neuronal (or glial) mass fraction, the ratio between glial and neuronal tissue mass, and the neuronal mass “allotted” to each glial cell are all a function of average glial cell mass, and can be estimated from the measured glial cell density by single power functions in any brain structure, and in any species.

## Discussion

Here we use chi-square minimization of a simple model that relates variations in glial and neuronal cell densities across brain structures and species to show that, in the absence of direct measurements of average neuronal and glial cell sizes, it is possible to estimate average neuronal and glial cell mass in a tissue from their respective densities. Average glial cell mass can be estimated from glial cell density (according to the equation ***m_g_*** = 1.648 ***d_gmes_***^−0.370^ pg) but not from neuronal density, and average neuronal cell mass can be estimated from neuronal cell density (according to the equation ***m_n_*** = 0.649 ***d_nmes_***^−1.004^ pg), but not from glial cell density. Our model allows us to estimate that average neuronal cell size varies tremendously across brain structures, and also across species for a given brain structure, between 0.264 pg in the cerebellum or 5.004 pg in the rest of brain and 124.051 pg in the cerebral cortex (that is, by a factor of 29× without the cerebellum, or over 500 × if the cerebellum is included, in the species examined here). In contrast, average glial cell size varies only modestly, around smaller values of between 3.454 and 5.619 pg (that is, by a factor of only 1.4 ×, or 2.0 × if the cerebellum is included). These estimates are consistent with the enormous diversity of neuronal cell types, which include local interneurons and projection neurons, in the face of only modest diversity of glial cell types, which are mostly local (Picker et al., 1981; Barres, 2008; Mishima and Hirase, 2010), although particular astrocyte types have been shown to vary significantly in size between small rodents and large primates, including humans (Oberheim et al., 2009). It is fundamental to keep in mind, however, that glial cell types are varied; astrocytes, oligodendrocytes, and microglial cells have different morphological characteristics and functions, and probably occur in different relative numbers in neural tissue. Astrocytes may not necessarily be the most numerous glial cell type; in the human cerebral cortical gray matter, for instance, astrocytes are only 20% of all glial cells, while oligodendrocytes account for 75% of glial cells (Pelvig et al., 2008). However, given the mathematical fact that the sum of individual power functions is not itself a power function, our findings that universal power functions apply to the glial cell composition as a whole in brain tissue, discussed below, suggest that similar mechanisms apply that regulate the addition of each glial cell type to the parenchyma. Thus, although we cannot at present discern between numbers of cells of each glial subtype, we can infer that the scaling rules described here must apply separately to each subtype.

### Comparison with the literature and practical implications

We have estimated that while neurons vary greatly in average cell mass, glial cells on average vary little in mass, both across brain structures and species. This is compatible with various accounts in the literature of similar glial densities but widely varying neuronal densities in the cerebral cortex across species as diverse as mice, humans, elephants and whales (Tower and Young, 1973; Haug, 1987), as we have observed in rodents (Herculano-Houzel et al., 2006), primates (Herculano-Houzel et al., 2007; Azevedo et al., 2009; Gabi et al., 2010) and eulipotyphlans (Sarko et al., 2009). The small degree of variation in glial cell mass that we predict is in agreement with recent findings in the literature comparing mouse and human astrocytes (Oberheim et al., 2009).

We emphasize that the estimated cell masses are not simply the inverse of the corresponding cell densities, but rather are proportional to a fixed power of cell density, with an exponent close to 1 for neurons, and a much smaller exponent for glial cells. The lack of linearity between glial cell mass and inverse glial cell density reflects the fact that, proportionally, the glial mass fraction decreases significantly with glial cell mass. It has been proposed recently that there is a universal neocortical reciprocal glial density of 1 glial cell per 0.047 nL of tissue (Carlo and Stevens, 2013). That proposal is flawed, however, as it disregards the small but highly consequential variation in glial cell density shown here, and fails to acknowledge the codependency of neuronal and glial cell densities, which are linked by the glial mass fraction of the tissue (see Equation 2). That calculation also disregarded a significant term inversely proportional to cortical thickness that should make the density vary systematically with cortical mass and order (Carlo and Stevens, 2013). Our model estimates a range of typically 5–15 pg of neuronal mass per glial cell, which increases together with small variations in average glial cell mass.

Applying our model to neuronal densities found in the literature, we can estimate the variation in neuronal density in a much broader range of species. For instance, with an average of 3661 neurons/mg in the cortical gray matter (Herculano-Houzel et al., 2014b), approximated to an inverse neuronal density of 273 pg/neuron, we estimate that the average neuron in the elephant cortical gray matter weighs 181 pg, in contrast to 34 pg for the average neuron in the human cortical gray matter estimated from a density of 19,541 neurons/mg in the tissue (Azevedo et al., 2009), 8 pg for the average neuron in the mouse cerebral cortex (gray and white matter), and only 4 pg for the average neuron in the cortical gray matter of the Etruscan shrew (from a neuronal density of 1,70,000 neurons/mm^3^; Stolzenburg et al., 1989). Moreover, the difference in estimates obtained for the combined gray and white matter and for gray matter alone allow us to infer that, of the estimated 48 pg that the average neuron in the human cerebral cortex weighs, only 14 pg, or less than a third of the cell mass, are on average located in the white matter. Thus, although the white matter represents 44% of the cortical mass (Azevedo et al., 2009), most of the mass of individual neurons is predicted to be localized in the cortical gray matter.

### Fundamental, universal properties of brain tissue

We show that, across brain structures and species, the large variation in the average neuronal cell size, ***m_n_*** is strongly and uniformly correlated to the G/N ratio ***N_g_/N_n_***, by an almost linear power law: ***N_g_/N_n_*** = **0.137(*m_n_***)^0.922^. The G/N ratio, however, is not strongly correlated with variation in average glial cell size, ***m_g_***. The correlation between ***m_n_*** and ***N_g_/N_n_*** is consistent with our hypothesis that variations in the latter are a consequence of variations in the former, that is, that the glia/neuron ratio in any brain tissue or species is determined by the average cell mass of the neurons in that tissue (Herculano-Houzel et al., 2006; Herculano-Houzel, 2011a). We propose that the relationship between the G/N ratio and average neuronal cell mass is a universal property of any brain tissue, achieved in a manner that will be developed further in the evo-devo model of brain tissue construction below.

While variations in ***m_g_*** are small, they have however significant consequences, as we find that these small variations define the glial and neuronal mass fractions of brain tissue, in what seems to be another universal property of any brain tissue. The neuronal and glial mass fractions of brain tissue can be visualized as the result of passing the tissue through a hypothetical sieve that separated all neurons from all glial cells (again, our model ignores the vascular component of the tissue, which is smaller than 5% of the tissue; Lawers et al., 2008; Karbowski, 2011). The sum of the masses of all neuronal cells constitutes the total neuronal mass of the tissue, which amounts to the product between the number of neurons and their average cell mass, ***m***_*n*_.***N**_n_*. The same applies to the sum of the masses of all glial cells in the tissue, which constitutes the total glial mass of the tissue and amounts to the product ***m_g_.N_g_***. The fraction ***m***_*n*_.***N***_*n*_/(***m***_*n*_.***N***_*n*_ + ***m_g_.N_g_*)** is the neuronal mass fraction ***f_n_*** of the tissue, that is, the fraction of tissue mass that is composed by neurons, and the complementary fraction, 1-***f***_*n*_, is the glial mass fraction ***f_g_*** of the tissue. Our model shows that all brain structures analyzed are mostly neuronal in their mass, with a neuronal mass fraction that ranges typically between 60 and 80%, while the glial mass fraction ranges between the complementary values of 20 and 40%, with no systematic difference between cerebral cortex, cerebellum, and rest of brain. Remarkably, variations in the glial mass fraction ***f_g_*** (and therefore also in the neuronal mass fraction ***f_n_***) are a universal function of variations in the average glial cell mass ***m_g_***, and do not correlate with variations in ***m_n_***, despite the much larger amount of variation in ***m_n_*** than in ***m_g_*** across brain structures and species. This indicates that the relationship between the neuronal (or glial) mass fraction and average glial (but not neuronal) cell mass, that is, the neuron/glia mass ratio (which is distinct from the G/N ratio), is another universal property of any mammalian brain tissue: a shared, universal function of ***m_g_*** (Figure 13).

The two universal properties related above are mathematically related to a third universal, fundamental characteristic of brain tissue, of great biological relevance: the neuronal mass per glial cell, which varies typically between 5 and 15 pg as a universal function of glial cell mass, ***m***_*g*_ (Figure 14) in a way such that to larger glial cells corresponds a larger neuronal mass ***m_n_.N_n_***. Interestingly, the smallest neuronal mass per glial cell, which is 3.8 pg, is only slightly larger than the average glial cell itself (3.5 pg), and the largest neuronal mass per glial cell, 20.1 pg, amounts to less than about 4x the corresponding average glial cell mass (5.5 pg). This implies that individual glial cells provide functional support to an amount of neuronal tissue that varies in mass from just slightly more than the glial cell itself to up to a few times its mass. Thus, the neuronal mass that is supported by each glial cell is a universal function of average glial cell size across brain structures and species alike. In fact, it can be demonstrated that the variation of the glial mass fraction ***f_g_*** of a tissue as a universal function of ***m_g_*** is algebraically related to the universal relationship between ***m_g_*** and ***m_n_.N_n_***.

### Functional and evolutionary implications

Across brains that vary in mass by over 100,000× and in numbers of neuronal and non-neuronal cells by over 10,000×, with widely varying neuronal densities and G/N ratios, here we describe two relationships that apply uniformly across brain structures and species, and thus appear to be fundamental properties of brain tissue. These uniform relationships imply that there are universal mechanisms that determine (1) the amount of neuronal tissue that is taken care of by each individual glial cell, and thus also the glial mass fraction (or its mathematical correlate, the neuron/glia mass ratio), and (2) the numeric G/N ratio. The first fundamental property depends on small variations of ***m_g_***, while the second depends on large variations of ***m_n_***.

It makes intuitive sense that each glial cell should provide functional support for a mass of neurons that is not much larger than the glial cell itself. We estimate that this neuronal mass per glial cell varies from about 1× to about 3× the average mass of the individual glial cells. In other words, the neuron/glia mass ratio varies typically between 2 and 3, as the glial mass represents between 20 and 40% of brain mass, while neuronal mass represents the complementary 60–80%.

It also makes physiological sense that larger individual glial cells should be able to support larger neuronal masses. But the relationship we describe here indicates an upper limit to how much neuronal mass a single glial cell can support, which also seems to make physiological sense, especially in view of the functions that glial cells perform, providing metabolic support, and ionic and neurotransmitter stability (Magistretti et al., 1999; Ullian et al., 2001; Fields and Stevens-Graham, 2002; Lee et al., 2012), and with mostly non-overlapping tiling of glial cells in the tissue (Bushong et al., 2002; Ogata and Kosaka, 2002; Nedergaard et al., 2003; Halassa et al., 2007; Hughes et al., 2013). Thus, we suggest that brain tissue, regardless of the structure, is constructed in a way that is limited by the extent of support that each individual glial cell can provide to neurons in the area, which in turn depends on the average mass of individual glial cells. As a consequence of the regulation of the amount of neuronal mass that is supported by individual glial cells, the neuron/glia mass ratio varies relatively little (between 1 and 3), and as a uniform function of average glial cell mass, even though brain structures vary so much in size, neuronal density, estimated average neuronal mass, number of cells and the numeric ratio between neurons and glial cells across species and from one structure to another within the same brain.

Assuming that the same universality in the determination of the neuron/glia mass ratio applies to how astrocytes occupy brain tissue (Nedergaard et al., 2003), and given their known role in synapse formation and functional support (Ullian et al., 2001), it is tempting to speculate that the universal determination of glial cell mass and glial mass fraction of brain tissue correlates with an also universal distribution of synapses in brain tissue, across different structures and species (Cragg, 1967; Schüz and Palm, 1989; Braitenberg and Schüz, 1998). We are currently working on a comparison of numbers of synapses across brain structures and species.

Here we show that, across 90 million years of evolution (Murphy et al., 2004) and 4 orders of magnitude, glial cells as a whole have been added in very much the same manner to major brain divisions and to 27 mammalian species belonging to 4 groups. In contrast, neuronal cells are added to different brain structures in various manners, depending also on the mammalian order that the species belongs to. Our finding indicates that either the rules that govern the addition of glial cells to the brain have changed in exactly the same way across all species examined here since their departure from the last common ancestor, or else these rules have been kept unchanged for at least 90 million years. The shared glial scaling rules are evidence that while neurons have been largely free to vary in morphology and physiology during evolution, strong selective pressures must have been keeping glial cell variability to a minimum, such that only a few different astrocyte and oligodendrocyte types are recognized (Walz, 2000; Barres, 2008), all the while maintaining very similar properties among mammals (Picker et al., 1981; Mishima and Hirase, 2010), and even in amphibia (Kuffler et al., 1966). Such evidence of strong selective pressure against glial cell variability implies that the functions exerted by these cells are so fundamental for brain physiology that they can hardly be modified without compromising brain viability, and therefore the survival of the individual. In line with this notion, dysregulation of glial cell function can contribute to diseases of the nervous system such as ALS (Nagai et al., 2007) and fragile X syndrome (Jacobs and Doering, 2010) and may be involved in the mechanism of general anesthesia (Thrane et al., 2012). Importantly, our data do not show that the average glial cell mass, glial mass fraction or neuron/glial mass ratio are constant, but rather that they vary little across brain structures and species—and in a uniform, predictable manner that depends on the very small variation in average glial cell mass in the tissue.

Although the neuron/glia mass ratio varies with ***m_g_***, it must however be achieved through a simultaneous regulation of the number of glial cells that will support the tissue. This is expressed as the glia/neuron ratio, which we have found to depend on the average mass of the neurons in the tissue. Our estimates thus indicate that the G/N ratio is determined by the average neuronal cell mass in the tissue, and it is not necessary to evoke other factors, such as neuronal activity or metabolic cost (Reichenbach, 1989; Herculano-Houzel, 2011b) to account for the G/N ratio. We have recently estimated that, contrary to expectations, the average metabolic cost per neuron does not increase together with neuronal cell size; indeed, the metabolic cost per neuron is calculated to remain fairly constant across neuronal cell sizes and species (Herculano-Houzel, 2011b). In this case, a larger metabolic cost incurred by larger neurons could not explain why they are uniformly accompanied by predictably larger proportions of glial cells. Instead, we propose that a simple, mechanical, evo-devo, economical self-organizing process accounts simultaneously for all of the uniform glial scaling rules found here as well as the non-uniform neuronal scaling rules. The G/N ratio is thus another fundamental property of the tissue, and rather than seeking activity-related factors that might influence it, we suggest that it is the G/N ratio that limits aspects of neuronal function. At the same time, the G/N ratio might simply reflect a basic property of brain tissue composition, which is the neuronal mass supported per individual glial cell, with both parameters being determined by variations in ***m_g_***. The next section explores how this regulation could be achieved in an evo-devo model of brain tissue construction.

### An evo-devo model of brain tissue construction

Two fundamental properties of brain tissue described here need to be accounted for, namely (1) a slowly-varying amount of neuronal tissue that is taken care of by each individual glial cell (and therefore the neuronal/glial mass ratio) depending universally on the average size of the glial cell and (2) a rapidly-varying numeric G/N ratio that depends universally on average neuronal cell size. We propose that those fundamental properties arise in a universal manner across brain structures and mammalian species as a result of large variations in numbers of neurons and average neuronal size, which are then matched by numbers of glial cells to match the resulting total neuronal mass, adjusted to small variations in average glial cell size. This results in brain structures that, while varying in numbers of neurons and total neuronal mass by several orders of magnitude, maintain a universal relationship between total neuronal and total glial mass.

The principal component analysis applied to our large dataset points to a first factor that loads with ***m_g_***, ***m_n_***, ***f_g_***, and ***N_g_/N_n_***. As discussed above, we find that ***f_g_*** varies as a universal function of ***m_g_***, while ***N_g_/N_n_*** varies as a universal function of ***m_n_***. This leaves only ***m_g_*** and ***m_n_*** as independent variables. The second factor to explain the biological variation in our dataset loads with ***N_n_***, ***N_g_***, and ***m_n_***.***N_n_***. However, ***N_g_*** varies as a function of ***m_n_***.***N_n_***, and this in turn depends simply on the additional determination of ***N_n_***. It thus transpires that only three variables are necessary to determine the cellular composition and final mass of any mammalian brain structure: average glial cell mass ***m_g_***, average neuronal cell mass ***m_n_***, and the number of neurons ***N_n_***, from which all other cellular properties of the tissue are derived. We next propose how this might occur in brain development and evolution.

**Figure 15:**
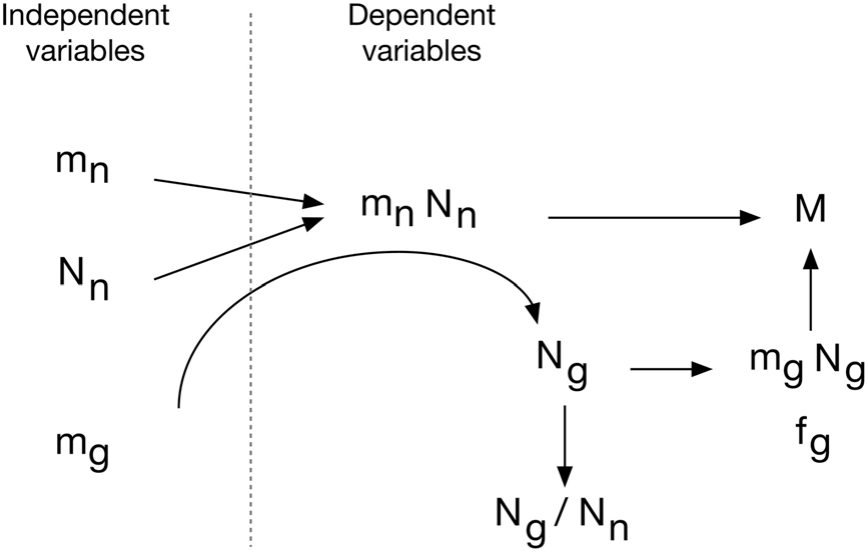
**Evo-devo model of brain tissue construction**. Independent variables are average neuronal cell mass (***m_n_***), average glial cell mass (***m_g_***) and number of neurons (***N_n_***). The product ***m_n_.N_n_*** is the total neuronal mass in the tissue, which is then invaded by a number of glial cells ***N_g_*** that depends on small variations in ***m_g_*** to create a total glial mass that matches the total neuronal mass in the tissue, depending on the ratio of neuronal cell mass per glial cell that we propose to be determined by ***m_g_***. This results in a certain neuronal mass fraction, ***f_n_***, and corresponding glial mass fraction, ***f_g_***. In evolution, variations in ***N_n_***, linked or not to variations in ***m_n_***, would yield structures of different masses whose glia/neuron ratios depend on ***m_n_*** and its ratio to ***m_g_***. Additionally, smaller variations in mg lead to variations in the glial mass fraction, ***f_g_***, and in the neuron/glial mass ratio.

The evo-devo model of brain construction that we propose, shown in Figure 15, is based on the fact that brain parenchyma is initially almost entirely neuronal in development (Brizzee et al., 1964; Bandeira et al., 2009). We propose that (1) the average cellular mass of neurons and glial cells is determined genetically in such a way that they are broadly and linearly correlated with each other across brain structures and species, but that also allows a small, independent variation in ***m_g_***. Next, (2) the neurons that compose the tissue are added in numbers that are related to ***m_n_*** in a tissue- and order-specific manner. Thus, these are the independent parameters in constructing brain tissue: ***m_g_***, ***m_n_***, and ***N_n_***.

The combination of numbers of neurons generated at a certain pre-determined average cell size yields the total neuronal mass of the tissue, ***m_n_.N_n_***. This neuronal mass is invaded by glial cell precursors, and these precursors generate glial cells of a predetermined average size ***m_g_*** and in a number ***N_g_*** that, depending on their average size, and through contact-dependent inhibition of precursor cell proliferation, yields a glial mass ***m_g_.N_g_*** to match the neuronal mass of the tissue. We propose that the matching of total glial to total neuronal mass is the mechanism that determines the total number of glial cells that will compose the tissue. This is consistent with the finding that ***N_g_*** is not universally related to either ***N_n_*** or ***m_n_***, but it is related universally to the product ***m_n_.N_n_***, that is, to total neuronal mass in the tissue, and even more strongly to the ratio ***m_n_***.***N_n_***/***m_g_***. In this scenario, therefore, the ratio between glia/neuron cell numbers is simply a result of this matching of glial to neuronal mass in the tissue, yielding a glia/neuron ratio that varies with ***m_n_***, but depends more precisely on the ratio ***m_n_/m_g_***. However, because ***m_n_*** is much more variable than ***m_g_***, and because (as per our hypothesis Equation 4) there is significant correlation between large variations in ***m_n_*** and small variations in ***m_g_***, the glia/neuron ratio also correlates strongly with ***m_n_***.

Mechanistically, we propose that the total glial mass that composes any brain tissue is achieved mostly through the regulation of glial cell proliferation, possibly by contact-dependent inhibition (Zhang and Miller, 1996; Hughes et al., 2013). In this case, small variations in ***m_g_*** should impact directly the numbers of glial cells required to achieve confluency in the tissue: A slightly smaller number of slightly larger glial cells will reach the same confluency attained by a slightly larger number of slightly smaller glial cells. Alternatively, the total glial cell mass could be defined directly by glial cell physiology, irrespective of contact-dependent population signals, depending rather on the interaction with neurons in the invaded parenchyma until a final equilibrium is reached at a certain neuronal to glial mass ratio. Knowing however that glial cell proliferation is subject to contact inhibition of progenitor cells (Zhang and Miller, 1996; Hughes et al., 2013), it seems more likely that the total glial mass in the tissue is matched to the total neuronal mass simply by the regulation of glial progenitor proliferation.

Our model is based simply on the independent variation of ***m_n_*** and ***N_n_*** (plus the residual variation in ***m_g_*** not accounted by the variation of ***m_n_*** and thus beyond the scope of our mathematical model). This nonetheless accounts very economically for brain development, brain structure diversity, and for evolutionary variations alike. In development, it explains how numbers of glial cells, the glial (or neuronal) mass fraction, glia/neuron ratio, and glial and neuronal densities are determined in a manner that is universal across brain structures and species, depending simply on the three original variables. Across brain structures, it explains how structure-specific average neuronal size and numbers of neurons could be determined (through genetic and other structure-specific factors controlling ***N_n_*** and ***m_n_***), and yet conform to the universal relationships between glia/neuron ratio and neuronal cell mass, and between glial mass fraction and glial cell mass. Likewise, large evolutionary variations in ***m_n_*** and ***N_n_*** with or without accompanying variations in ***m_g_*** would lead to large changes in neuronal mass and adult brain structure mass while maintaining the glia/neuron numeric ratio determined universally by ***m_n_***, and the neuronal/glial mass ratio determined universally by ***m_g_***.

We thus propose that the universal relationship between the amount of glial mass that accompanies a unit of neuronal mass reported here, which we call the fundamental building block of brain tissue, is a consequence of a universal mechanism whereby numbers of glial cells are added to the neuronal parenchyma during development, irrespective of whether the neurons composing it are large or small, but depending on the average mass of the glial cells being added.

### Conflict of interest statement

The authors declare that the research was conducted in the absence of any commercial or financial relationships that could be construed as a potential conflict of interest.
